# Purkinje Cell Activity Determines the Timing of Sensory-Evoked Motor Initiation

**DOI:** 10.1016/j.celrep.2020.108537

**Published:** 2020-12-22

**Authors:** Shinichiro Tsutsumi, Oscar Chadney, Tin-Long Yiu, Edgar Bäumler, Lavinia Faraggiana, Maxime Beau, Michael Häusser

**Affiliations:** 1 Wolfson Institute for Biomedical Research and Department of Neuroscience, Physiology and Pharmacology, University College London, London, UK

**Keywords:** purkinje cells, simple spikes, complex spikestwo-photon imaging, optogenetics, neuropixels, cerebellum, multisensory, timing, motor initiation

## Abstract

Cerebellar neurons can signal sensory and motor events, but their role in active sensorimotor processing remains unclear. We record and manipulate Purkinje cell activity during a task that requires mice to rapidly discriminate between multisensory and unisensory stimuli before motor initiation. Neuropixels recordings show that both sensory stimuli and motor initiation are represented by short-latency simple spikes. Optogenetic manipulation of short-latency simple spikes abolishes or delays motor initiation in a rate-dependent manner, indicating a role in motor initiation and its timing. Two-photon calcium imaging reveals task-related coherence of complex spikes organized into conserved alternating parasagittal stripes. The coherence of sensory-evoked complex spikes increases with learning and correlates with enhanced temporal precision of motor initiation. These results suggest that both simple spikes and complex spikes govern sensory-driven motor initiation: simple spikes modulate its latency, and complex spikes refine its temporal precision, providing specific cellular substrates for cerebellar sensorimotor control.

## Highlights

• A multisensory association task engages the cerebellum to drive timed behavior
• Simple spikes determine whether and when to initiate sensory-evoked motor actions
• Complex spike signals show highly conserved alternating parasagittal organization
• Acquired complex spike coherence contributes to precisely timed motor initiation

## Graphical Abstract

**Figure:**
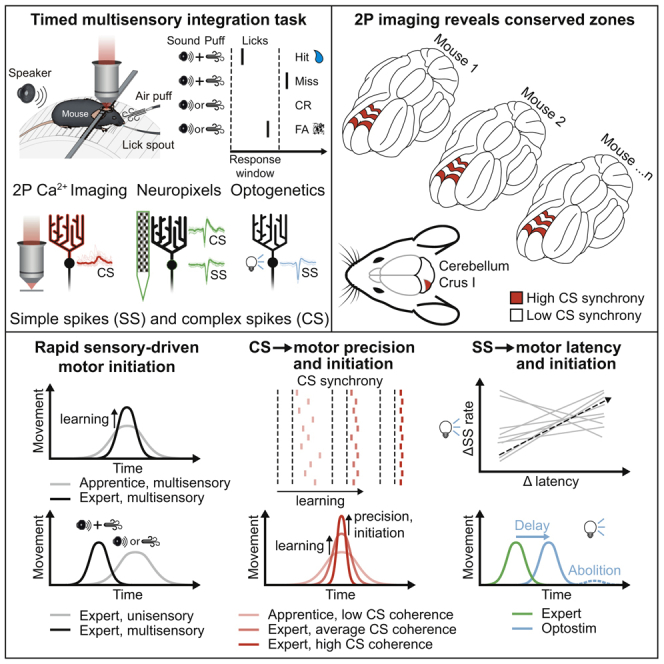

## Teaser

Tsutsumi et al. combine Neuropixels recordings, optogenetics, and two-photon imaging of Purkinje cells in the lateral cerebellum in a multisensory association task to reveal that short-latency simple spikes determine the initiation of sensory-evoked actions and its timing, whereas spatially organized and acquired coherence in complex spike signals contribute to its temporal precision.

## Introduction

Precisely timed initiation of motor actions in a rapidly changing environment is essential for survival. Optimal execution of these actions requires linking sensory integration and timed motor initiation. The cerebellum is well positioned to participate in this process, because it receives sensory and motor information from the periphery and from neocortical sources. This information is conveyed to the cerebellar cortex via two routes: the mossy fiber-granule cell-parallel fiber pathway and the inferior olive-climbing fiber pathway. These pathways converge onto Purkinje cells, where their inputs modulate spontaneous simple spikes and complex spikes, respectively. It is known that whisker-related neocortical sensory and motor streams converge on lobule Crus I (Proville et al., 2014) in the lateral cerebellum, which communicates with higher-order brain areas (Dum et al., 2002) and is particularly important in active sensory processing for purposeful behavior (Bower, 1997; Gao et al., 1996). This suggests that Crus I is an ideal target for understanding how sensory and motor representations are linked.

Simple spikes are known to represent both sensory and motor information. Sensory inputs can be integrated in cerebellar granule cells (Arenz et al., 2008; Huang et al., 2013; Ishikawa et al., 2015), which in turn activate Purkinje cells, resulting in bidirectional simple spike modulation (Mano and Yamamoto, 1980). Recordings from Purkinje cells during reflexive whisking have revealed alternating decreases and increases in simple spike probability in response to whisker pad stimulation, amplifying reflex whisking (Brown and Raman, 2018). Decreases in simple spike firing in response to conditioned sensory stimuli are learned and crucial for the conditioned response during eye-blink conditioning (Jirenhed et al., 2007). Moreover, changes in simple spike rate precisely lead and mirror motor kinematics (Brown and Raman, 2018; Herzfeld et al., 2018), suggesting a role in sensory-evoked motor initiation and its timing. How these heterogeneous simple spike patterns contribute to active sensorimotor associations remains unclear.

Climbing fiber inputs to Purkinje cells play a role in motor timing (Llinás, 2011; Welsh, 2002; Welsh et al., 1995): synchronous climbing fiber inputs to Purkinje cells result in synchronous complex spikes, which are dynamically organized during voluntary (Welsh et al., 1995) and sensory-evoked movements (Welsh, 2002). Ablation of climbing fiber inputs disrupts the timing of cued licking, lever pushing, and lever press-tongue coordination (Welsh, 2002). Complex spike synchrony is organized into parasagittal bands of Purkinje cells projecting to distinct sets of nuclear neurons (Apps and Hawkes, 2009; Sugihara, 2011), evoking a transient hyperpolarization followed by rebound firing (Bengtsson et al., 2011; Tang et al., 2019), which can drive behavior (Gao et al., 2018; Ten Brinke et al., 2017; Witter et al., 2013). In addition, other studies have also demonstrated a role of climbing fiber inputs in sensory processing (Brown and Raman, 2018; Gaffield et al., 2019; Ju et al., 2019; Knogler et al., 2019; Ten Brinke et al., 2019). However, the way in which sensory-evoked climbing fiber inputs realize associative and timed motor initiation remains unclear.

Here we combined a multisensory association task, Neuropixels recordings of both simple and complex spikes, optogenetic manipulation of simple spikes, and two-photon calcium imaging of complex spike signals to study the role of Crus I Purkinje cells in sensorimotor behavior. Our findings indicate that both simple spikes and complex spikes contribute to the initiation of sensory-driven behavior, in which simple spikes can modulate the timing of initiation and coherent complex spikes sharpen its temporal precision.

## Results

### A Multisensory Association Task for Probing the Role of the Cerebellum in Rapid Sensory-Driven Behavior

**Figure 1:**
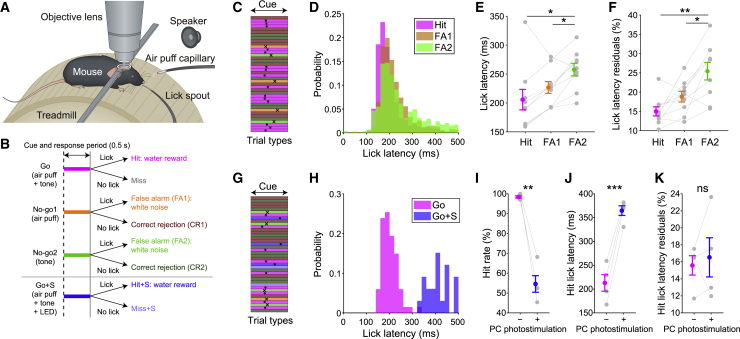
A Multisensory Association Task for Probing the Role of the Cerebellum in Rapid Sensory-Driven Behavior (A) Schematic of the multisensory association task and experimental setup. (B) Task structure. Air puff + tone (Go, magenta), air-puff-only (No-go 1, orange), and tone-only (No-go 2, green) trials were provided. During optogenetics experiments (under gray line), Purkinje cells were photostimulated in a subset of Go trials (Go+S, blue). (C) Representative performance in a subset of a single session. Colors represent trial types. Dots represent first licks for Hit trials. Crosses represent first licks for FA trials. (D) Lick latency distribution for Hit, FA1, and FA2 trials pooled across animals. (E) Lick latency from cue onset across trial types. (F) Same as (E) but for lick latency residuals. (G) Same as (C) but during optogenetics experiments. (H) Lick latency distribution after Go cues in the presence (Go+S) or absence (Go) of photostimulation in a representative session. (I) Hit rate in the absence (−) or presence (+) of LED photostimulation. (J) Same as (I) but for lick latency in Hit trials. (K) Same as (I) but for lick latency residuals in Hit trials. See also Figure S1 and Table S1.

We designed a multisensory association task that allows two-photon imaging, Neuropixels recordings, and optogenetic stimulation in head-fixed mice (Figure 1A). In this task, water-restricted mice were trained to rapidly discriminate between either a tone or an air puff to the whisker pad (unisensory cues) and the two sensory stimuli presented together (multisensory cues). Licking to the lick port was rewarded with sugar water if it occurred within a 500 ms window of the multisensory cues (Go cue − Hit trials) (Figure 1B and 1C). Licking to either individually presented stimulus, the air puff (No-go 1 cue − False alarm 1 [FA1] trials) or the tone (No-go 2 cue − False alarm 2 [FA2] trials), was punished with white noise. The absence of licking during the 500 ms window, in either trial, was neither rewarded nor punished (Go cue − Miss trials; No-go 1/2 cue − Correct rejection 1/2 [CR1/2] trials). This task therefore requires a close linkage between sensory integration and timed motor initiation. In expert animals (d′ > 2.5), the lick latency was shorter for air-puff-driven licking (Hit and FA1) than tone-driven licking (FA2) (Figures 1D and 1E; Table S1). However, the lick latency after the multisensory stimulus (Hit) was indistinguishable from that after the air-puff-only stimulus (FA1). We observed that the air-puff-driven first licks (Hit and FA1) were more precisely timed than the tone-driven ones (FA2) (Figures 1D and 1F; Table S1). However, the temporal precision of the first licks after multisensory stimuli (Hit) was not different from that after air-puff-only stimuli (FA1). These observations suggest that this task promotes acquisition of precisely timed motor initiation in response to multisensory stimulation.

### Optogenetic Cerebellar Stimulation Disrupts Sensory-Driven Motor Initiation

To test the cerebellar contribution to this task, we performed targeted optogenetic manipulation of simple spike firing in Purkinje cells in Crus I by using a Pcp2-Ai32 line (Nguyen-Vu et al., 2013) expressing channelrhodopsin-2 (ChR2) in all Purkinje cells. A light-emitting diode (LED) was used for unilateral delivery of blue light to Crus I, synchronized to Go cue presentation (Figure 1B). Photostimulation of Purkinje cells during the Go cue significantly reduced the Hit rate (n = 4 mice; 98% ± 1% versus 55% ± 8%, p = 0.0023) (Figure 1I), suggesting the involvement of Crus I in motor initiation. Moreover, the latency of the first lick in the lick bout that was initiated during optogenetic stimulation of Purkinje cells was substantially delayed in a representative session (Figures 1G and 1H). Mice exhibited longer lick initiation latencies in Hit trials with photostimulation (Hit+S) than in non-photostimulation Hit trials (n = 4 mice; 210 ± 30 versus 360 ± 20 ms, p = 9.1 × 10^−4^) (Figure 1J). However, the lick latency residuals remained constant (n = 4 mice; 16% ± 2% versus 17% ± 5%, p = 0.70) (Figure 1K), suggesting that optogenetic modulation of simple spikes has a minimal effect on temporal precision of the latency of sensory-driven motor initiation.

Because photostimulation substantially delayed lick initiation, we sometimes observed first licks slightly offset from the 500 ms response window of our task (Figure S1A), so we extended our analysis window to 1,000 ms. Lick latency remained significantly delayed by photostimulation (n = 4 mice; 210 ± 40 versus 420 ± 20 ms, p = 0.0025) (Figure S1B) and the temporal precision of lick latency remained similar regardless of photostimulation (n = 4 mice; 16% ± 2% versus 22% ± 8%, p = 0.25) (Figure S1C).

We next asked whether photostimulation could abolish or simply delay lick initiation beyond the initial 500 ms analysis window. To dissociate these two possibilities, we separately compared the number of out-of-window (500–1,000 ms) first licks and abolished first licks on Go and photostimulated Go trials. Photostimulation resulted in increased numbers of both delayed (n = 4 mice; 0.3% ± 0.4% versus 15% ± 7%, p = 0.029) (Figure S1D) and missed (n = 4 mice; 1.2% ± 0.8% versus 31% ± 6%, p = 0.0039) (Figure S1E) first licks, consistent with an important role for Crus I Purkinje cell simple spikes in both motor initiation and its timing.

The effects on Hit rate and lick latency were not observed in wild-type animals in which identical LED illumination was provided (n = 3 mice; Hit rate: 95% ± 5% versus 98% ± 1%, p = 0.58; Go lick latency: 170 ± 20 versus 159 ± 8 ms, p = 0.52) (Figures S1F–S1J), suggesting that the LED light itself did not affect the behavior.

To identify whether these effects on licking resulted from a non-specific motor perturbation, we randomly provided 500 ms optogenetic stimulation in Pcp2-Ai32 mice performing continuous spontaneous licking. Photostimulation did not disrupt spontaneous licking (ratio of licking epochs; n = 4 mice; 100% ± 0% versus 95% ± 6%; p = 0.25) (Figures S1K–S1M). Moreover, the sensory-evoked lick rate remained unchanged regardless of the presence of photostimulation once initiated (Hit versus Hit+S; n = 72 and 30 trials; 7 ± 1 versus 7 ± 2 licks/s; p = 0.38) (Figures S1N and S1O).

Overall, these results suggest that in this task, physiological simple spike firing in Crus I Purkinje cells is crucial for the rapid initiation of sensory-driven motor actions, but not for the motor actions per se.

### Heterogeneous Sensorimotor Representations in Purkinje Cell Simple Spikes

**Figure 2:**
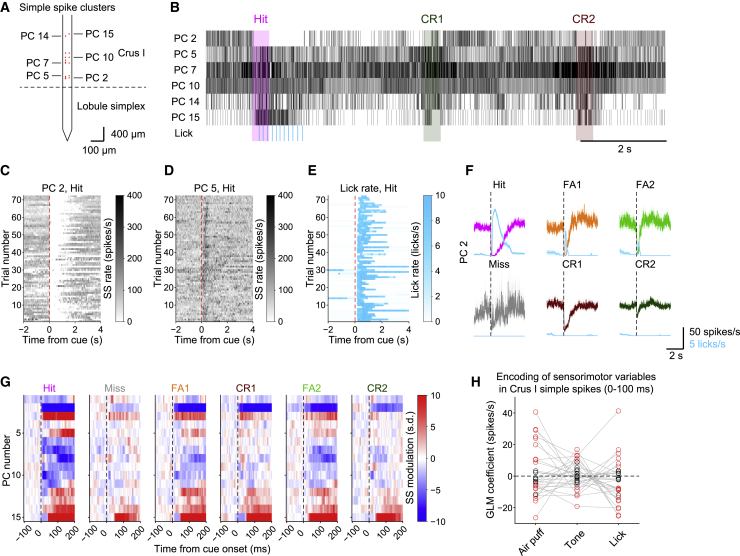
Heterogeneous Sensorimotor Representations in Purkinje Cell Simple Spikes (A) Simultaneously recorded Purkinje cells (PCs, red dots) on a Neuropixels probe. The putative boundary between lobules is indicated by a dashed line. (B) Representative raster plots of simple spikes (black) and licks (light blue) during the task. Note the pause and increased simple spike firing during licking. Shaded areas represent the duration of sensory stimuli (500 ms) for the corresponding trial type. (C) Single-trial simple spike firing rate during Hit trials from a representative Purkinje cell (PC 2). (D) Same as (C) but for PC 5. (E) Single-trial lick rate during Hit trials. (F) Trial-averaged simple spike firing rate (n = 72, 33, 29, 9, 67, and 90 trials for Hit, FA1, FA2, Miss, CR1, and CR2) aligned to the sensory cue onset from PC 2 overlaid by the trial-averaged lick rate for each trial type. (G) Spatially aligned heatmaps for trial-type-averaged simple spike firing rate from clusters in (A). Simple spike firing rate of −100 to 200 ms time window from the onsets of sensory stimuli are *Z* scored and color coded for each cell per trial type. (H) GLM coefficients for the air puff, tone, and lick initiation fit to simple spike modulation at 0–100 ms from the onset of sensory stimuli in individual Crus I PCs. Gray lines represent individual cells. Red circles represent significant contributions of the predictors to the model, and black circles represent non-significant ones. See also Figures S2 and S3 and Table S1.

To further examine the role of Purkinje cell simple spikes in our task, we performed Neuropixels probe recordings (Jun et al., 2017; Kostadinov et al., 2019) in Crus I of well-trained (d′ > 2.0) Pcp2-Ai32 mice (n = 4) (Figures S2A–S2F). Simple spikes could be simultaneously recorded from many Purkinje cells in each animal (Figure 2A). Simple spike firing rate from all recorded Purkinje cells across lobules was 84.1 ± 43.7 spikes/s (n = 4 mice, 4 sessions, and 64 clusters) (Figure S2H), consistent with previously reported values from Crus I (Bryant et al., 2010). Simple spike raster plots revealed both positive and negative changes in Crus I Purkinje cell simple spike firing during the task: some Purkinje cells exhibited decreases in simple spike firing after sensory stimuli and during licking, and others increased firing (Figure 2B). Individual Purkinje cells showed consistent task-related modulation during Hit trials for a given session (Figures 2C and 2D), largely reflective of licking (Figure 2E). Individual Purkinje cells showed a mixture of both sensory and motor-related simple spike modulation: trials with licking (Hit, FA1, and FA2) (Figure 2F, top row) showed larger modulation of simple spikes than those without licking (Miss, CR1, and CR2) (Figure 2F, bottom row). We observed that most simultaneously recorded Crus I Purkinje cells showed either task-related activation or suppression (Figure 2G).

To dissociate the simple spike representations of the air puff, tone, and licking, we fit a generalized linear model (GLM) to the changes in simple spike rate during the initial 100 ms after the onset of sensory stimuli by using the presence (1) or absence (0) of the air puff, tone, and licking (Figure 2H). We chose a window of 0–100 ms from sensory cue onset, because no licks were observed in this period; therefore, it could not be influenced by the lick-related movement execution or sensory feedback. We found that most recorded Crus I Purkinje cells (25 of 30 cells) showed significant (p < 0.05) simple spike modulation by the air puff, tone, or licking (red dots in Figure 2H). The task representation within individual Crus I Purkinje cells was highly heterogeneous across the population; various mixtures of positive and negative simple spike modulation associated with the air puff, tone, or licking (Figure 2H). However, in our task design, the licking representation could involve both multisensory perception and lick initiation, because they are temporally intermixed.

To distinguish among the preceding possibilities, we aligned the simple spike rate to the onset of both sensory-driven and non-task-related spontaneous lick bouts (Figures S3A and S3B), because the former includes multisensory discrimination but the latter does not. We fit the GLM to lick-onset-aligned pre-licking simple spike firing (−100 to 0 ms from the lick bout onset) by using the presence or absence of the air puff, tone, and licking as predictors (Figure S3C). We then compared the GLM coefficients for sensory-driven lick initiation with the raw pre-licking simple spike modulation for spontaneous lick initiation (−100 to 0 ms from the lick bout onset) (Figure S3D). We found that the representation of lick initiation in simple spike modulation was indistinguishable across the contexts. These results indicate that the observed pre-licking simple spike modulation mostly represents lick-initiation-related activity rather than multisensory-perception-related activity.

### Simple Spike Modulation in Crus I Contributes to Sensory-Driven Lick Initiation and Its Timing

**Figure 3:**
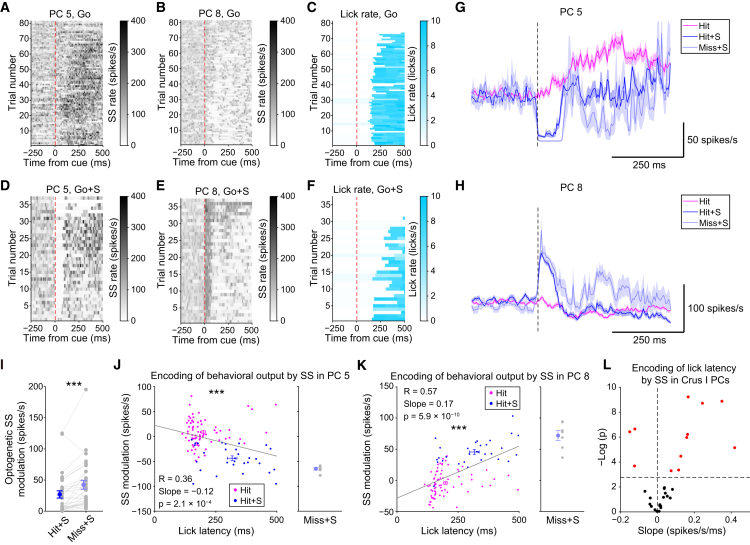
Simple Spike Modulation Contributes to Sensory-Driven Lick Initiation and Its Timing (A) Single-trial simple spike (SS) firing rate after Go cues in the absence of optogenetic stimulation from PC 5 (from Figure 2). The red dotted line represents the onset of the sensory cue. (B) Same as (A) but for PC 8 (from Figure 2). (C) Single-trial lick rate after Go cues without optogenetic stimulation. (D) Same as (A) but for Go cues in the presence of optogenetic stimulation (Go+S). (E) Same as (D) but for PC 8. (F) Same as (C) but for Go+S. (G) Trial-averaged SS rate of PC 5 during Hit trials, Hit + photostimulation (Hit+S), and Miss + photostimulation (Miss+S). A dotted line represents the onset of the sensory cue. (H) Same as (G) but for PC 8. (I) Optogenetically induced absolute changes in trial-averaged SS modulation at 0–100 ms from the onset of sensory stimuli during Hit+S and Miss+S trials compared with that during Hit trials. (J) Linear regression of single-trial lick latency to SS modulation of PC 5 during Hit and Hit+S trials. The SS modulation for Miss+S trials is shown on the right. (K) Same as (J) but for PC 8. (L) Volcano plot for the slope of the linear regression and the significance level individually fit for each PC in Crus I. A horizontal dotted line represents the Bonferroni-corrected threshold p value for significance (p = 0.0017). Red dots represent PCs with significant correlation with lick latency. See also Figure S2 and Table S1.

To test the role of simple spikes in lick initiation, we made Neuropixels recordings during optogenetic stimulation (Figure S2I). We observed both increases and decreases in simple spike firing rate in response to optogenetic stimulation (Figures 3A and 3B versus 3D and 3E), with the degree of optogenetic perturbation being correlated with the degree of reduction in licks (Figure 3C versus 3F). When grouping photostimulated trials into Hit (Hit+S) and Miss (Miss under photostimulation [Miss+S]) trials, optogenetic simple spike disruption at 0–100 ms latency was exaggerated during Miss+S compared with Hit+S in the representative cells (Figures 3G and 3H) and across all Crus I Purkinje cells (n = 30 cells from 4 mice; 27 ± 32 versus 42 ± 39 spikes/s for Hit+S and Miss+S, p = 2.8 × 10^−4^) (Figure 3I).

To test the role of simple spikes in timing, we performed linear regression between the short-latency simple spike modulation and the lick latency in these trials. We found that a subset of Crus I Purkinje cells exhibited a correlation between the simple spike modulation and the lick latency (Figures 3J and 3K). Across the population, the effect of simple spike modulation on lick latency was also bidirectional, but positive correlations were more prevalent (9 cells positive versus 3 cells negative out of 30 cells) (Figure 3L): in other words, in most Crus I Purkinje cells, an increase in simple spike rate delays lick initiation.

Altogether, these results indicate that sensory-evoked short-latency simple spike modulation in Crus I Purkinje cells influences the associated motor initiation and its timing.

### Spatiotemporal Task Representations in Complex Spike Signals

**Figure 4:**
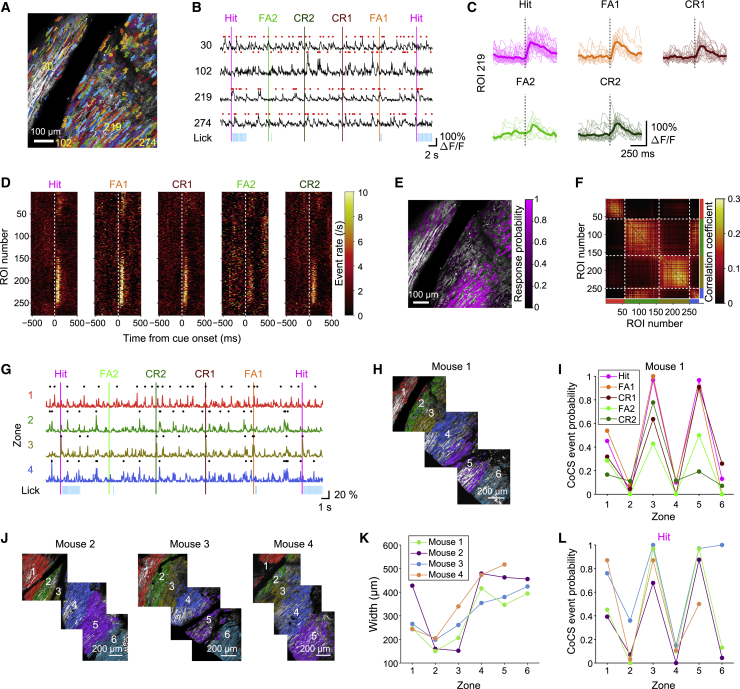
Task-Related Complex Spike Signals Are Organized into Conserved Alternating Parasagittal Stripes (A) Two-photon imaging field of view showing extracted Purkinje cell dendritic regions of interest (ROIs, pseudo-colored). (B) Representative fluorescence traces from selected ROIs (indicated by yellow numbers in A; ROIs are numbered from lateral to medial). Red dots represent extracted complex spike (CS) events. Vertical lines represent the sensory cue onset, and their colors represent corresponding trial types. (C) Single-trial fluorescence traces from a single ROI (ROI 219 in A and B) aligned to the onset of sensory stimuli (dotted lines) for each trial type. Thin lines represent single trials, and thick lines represent trial averages. (D) Trial-type-averaged CS event rate heatmap of ROIs in (A). White dotted lines represent the onset of sensory stimuli. (E) ROIs colored based on CS event probability for 0–250 ms from the onset of sensory stimuli during Hit trials. (F) Correlation matrix of fluorescence traces for a whole imaging session, clustered and sorted based on correlation similarity. White dotted lines represent boundaries of zones. Zonal identity is represented by the thick colored lines at the bottom and right (zone 1, red; zone 2, green; zone 3, yellow; zone 4, blue). (G) Co-activation traces from zones 1–4. Dots represent extracted co-activated complex spike (CoCS) events. (H) Spatial arrangement of three imaging fields across the expanse of Crus I. Six zones are identified and numbered from lateral to medial (zones 1–6). (I) CoCS event probability of zones 1–6 across trial types from a representative animal. (J) Assignments of functionally defined zones in Crus I across fields of view and across mice. (K) Widths of zones 1–6 from all mice. (L) Same as (K) but for CoCS event probability during Hit trials. See also Figures S4 and S5.

To examine the contribution of task-related complex spike synchrony to the behavior, we performed two-photon calcium imaging of Crus I Purkinje cell dendrites (Figure 4A) to monitor complex spike signals (Ozden et al., 2009; Schultz et al., 2009). Large global dendritic calcium signals, corresponding to complex spikes (Kitamura and Häusser, 2011) occurred at 1.24 ± 0.20 events/s (n = total 3,567 dendrites from 12 fields of view in 4 mice) (red dots in Figure 4B). Direct electrophysiological recordings of complex spikes in Purkinje cells using Neuropixels probes revealed similar mean rates (1.48 ± 0.33 spikes/s; n = 87 Purkinje cells) (Figure S2H), validating our complex spike detection from imaging data. Complex spike signals were time-locked to the onset of sensory cues (Figure 4C). We spatially sorted all the dendrites from lateral to medial and aligned their complex spike events to the onset of sensory cues (Figure 4D). We then mapped the probability of the complex spike events with a 0–250 ms latency from cue onset during Hit trials onto the imaging field of view (Figure 4E). This revealed alternating parasagittal stripes of responding and non-responding Purkinje cells. We also plotted the correlation matrix of anatomically sorted Purkinje cell dendrites by using spontaneous activity from inter-trial intervals when the activity did not reflect task-related events (Figure 4F). Here, we could identify highly correlated clusters of Purkinje cell dendrites, which almost precisely matched with alternating clusters of task-responsive and non-responsive dendrites (roughly regions of interest [ROIs] 1–50, 50–150, 150–250, and 250–280) (Figures 4D and 4F), thereby highlighting the innervation from both functionally similar and synchronous climbing fibers (Kostadinov et al., 2019). Neuropixels probe recordings of complex spikes from Crus I showed similar task-related responses and spontaneous correlations (Figures S4A–S4D), validating our event detection from imaging data. These results indicate that task-related complex spike signals are organized into alternating parasagittal stripes within which spontaneous complex spike signals are highly correlated.

### Task-Related Complex Spike Signals Are Organized into Conserved Alternating Parasagittal Stripes

Because complex spike signals are synchronized within anatomically defined zones at single-cell resolution (Tsutsumi et al., 2015), we sorted the correlation matrices using k-means clustering (Ozden et al., 2008) to functionally delineate regions of synchrony in Crus I, revealing regions within which complex spike signals were highly correlated (Figure 4F). The average width of these regions was 333 ± 41 μm (n = total 23 regions from 12 imaging fields of view from 4 mice) (Figures 4J and 4K), consistent with the widths of anatomically defined zones (Sugihara and Quy, 2007). Our overlapping fields of view enabled identification of these zones across fields of view within a single mouse (Figure 4H). We identified 6 zones spanning the entire Crus I and numbered them from lateral to medial (zones 1–6) (Figure 4H). Similar zonal structures were observed in the neighboring lobule simplex (Figure S5A). In this lobule, the overall alternating organization was similar to that in Crus I, but the response properties of the complex spike signals were slightly different (more tone responses) (Figures S5B and S5C), suggesting of lobule-specific functional specialization (Heffley and Hull, 2019). Neuropixels recordings of complex spikes also revealed task-related activity mostly in Crus I, with synchronous activity patterns (at 1 ms resolution) corresponding to the individual lobules (Figures S4A–S4D), thus validating our imaging results.

To extract the synchronous complex spike events within the zones, we identified co-activation events (Ozden et al., 2012) representing coherent complex spike signals within individual zones in Crus I (co-activated complex spike [CoCS] events > mean + 3 SD co-activation from an entire trace) (black dots in Figure 4G). We extracted task-related coherent complex spike responses (0–250 ms latency) within each zone and found that the probability of coherent complex spike signals was organized into an alternating fashion: zones 1, 3, and 5, but not zones 2, 4, and 6, were highly responsive to the task (Figure 4I). Surprisingly, we found that these zones were both anatomically and functionally conserved across animals: their spatial location (Figure 4J), widths (Figure 4K), and response probability in Hit trials (Figure 4L) almost matched across mice.

Altogether, these findings reveal that coherent complex spike signals in Crus I are functionally organized into highly conserved zones.

### Coherent Complex Spike Signals in Alternating Zones Represent Sensory Saliency

How do coherent complex spike outputs from the zones represent sensory aspects of the task? In our task paradigm, the air puff led to quicker and temporally more precise first licks than the tone (Figure 1), indicating that the former was more salient for mice than the latter. Therefore, we reasoned that the zonal complex spike output is larger for the air puff than the tone if these signals represent sensory saliency.

**Figure 5:**
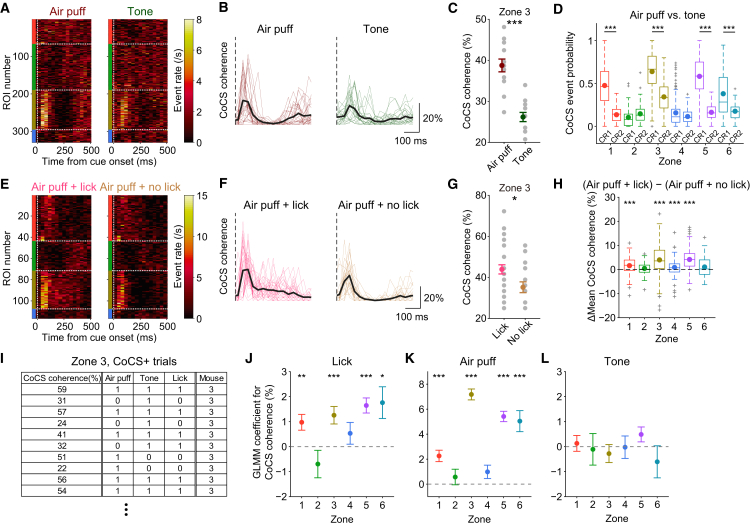
Enhancement of Coherence in Sensory-Evoked Complex Spike Signals Contributes to Motor Initiation (A) Trial-type-averaged CS event rate heatmap of ROIs for non-licking air puff (CR1) and tone (CR2) trials, from an example session. Vertical dotted lines represent the sensory cue onset, and horizontal lines represent zonal boundaries. (B) Single-trial co-activation traces of zone 3 for CR1 and CR2 trials in (A). Thin colored lines represent single trials, and thick lines represent trial averages. Vertical dotted lines represent the cue onset. (C) Comparison of coherence level within co-activation (CoCS) events with a 0–250 ms latency for CR1 and CR2 trials in (B). (D) Probability of CoCS events in zones 1–6 during CR1 and CR2 trials in single sessions pooled across mice. (E) Same as (A) but comparing licking trials and non-licking trials after sensory stimuli containing air puff stimulation. (F) Same as (B) but for licking trials and non-licking trials after sensory stimuli containing air puff stimulation. (G) Comparison of coherence level within CoCS events with a 0–250 ms latency for Hit+FA1 and Miss+CR1 trials in (F). (H) Differences in coherence levels of CoCS events in zones 1–6 across licking trials and non-licking trials after air puff stimulation (Hit+FA1 − Miss+CR1) in single sessions pooled across mice. (I) Example table of values used for the GLMM analysis. Rows represent individual trials with CoCS events. (J) GLMM coefficient for lick initiation fit to the trial-by-trial coherence level of CoCS events for each zone. (K) Same as (J) but for air puff stimuli. (L) Same as (J) but for tone stimuli. See also Figures S4, S6, and S7 and Table S1.

To test this possibility, we compared zonal complex spike responses during air-puff-only (CR1) and tone-only (CR2) non-licking trials. We observed that air-puff-only stimuli (CR1) evoked stronger complex spike signals than tone-only stimuli (CR2) in zone 3 (Figure 5A). In this zone, the coherence level of complex spike signals within co-activation events was higher during air puffs than tones (Figures 5B and 5C). Moreover, the air puff was associated with more CoCS events than the tone at the level of individual alternating zones (zones 1, 3, and 5) (Figure 5D; Table S1). Zone 6 also showed a similar trend. Accordingly, we could not distinguish between the zonal complex spike responses for multisensory stimuli and those for air-puff-only stimuli (Miss versus CR1 and Hit versus FA1) (Figures S6A–S6F; Table S1). These results indicate that sensory saliency is represented by zonal complex spike outputs in Crus I.

### Enhancement of Coherence in Sensory-Evoked Complex Spike Signals Contributes to Motor Initiation

To test whether sensory-evoked changes in complex spike coherence contribute to motor initiation, we compared the Crus I complex spike signals during air puff trials with or without licking (Air puff + lick versus Air puff + no lick; Hit+FA1 versus Miss+CR1) (Figure 5E). In zone 3, single-trial complex spike coherence within co-activation events was higher for licking trials than non-licking trials (Figures 5F and 5G). Accordingly, a difference in the coherence level of co-activation events between licking and non-licking trials was significant within individual alternating zones (zones 1, 3, and 5) (Figure 5H; Table S1). Zone 4 also showed a similar trend. These results indicate that more complex spikes are co-activated upon motor initiation in response to sensory cues than the sensory cues alone. In parallel, Neuropixels recordings revealed that the coherence level within CoCSs at 1 ms resolution tended to be enhanced upon lick initiation (n = 13 Purkinje cells, 47 and 40 trials from a single mouse; 28% ± 6% versus 26% ± 5%; p = 0.11) (Figure S4E). We also observed higher peaks in pairwise correlations between complex spike firing in Crus I Purkinje cells during Hit trials compared with CR1 trials (n = 13 cells; 5 ms bins) (Figures S4F–S4H), supporting the imaging results.

To make a direct comparison between licking and non-licking trials after multisensory stimuli (which was difficult because of a low Miss trial rate), we attenuated the tone level for the multisensory cue (Go cue) in a small subset of trials, thereby artificially increasing the number of Miss trials. Lick responses to the multisensory cue were accordingly reduced (Figure S6G). Lick initiation to the same multisensory stimuli (with a 58 dB tone) increased complex spike signals in zone 1 (Figures S6H and S6I). Comparisons across animals and zones did not reveal a significant enhancement of complex spike coherence, probably because of the small sample size (n = 5, 5, 7, 9, and 5 imaging sessions from 4 mice; p = 0.14, 0.45, 0.37, 0.10, and 0.26 for zones 1–5) (Figure S6J; Table S1); nevertheless, effect sizes were large (1.3, 0.5, 0.4, 0.8, and 1.4 for zones 1–5) (Table S1).

To tease apart the contributions of different behavioral features and account for confounding factors associated with the inconsistency in trial numbers across trial types, we built a generalized linear model with mixed effects (GLMM) for individual zones by using the air puff, tone, and lick initiation as predictors of the single-trial coherence level of complex spike signals (Figure 5I). We included the identity of individual mice as a mixed effect to control for variance across mice. We found that lick initiation significantly contributed to the single-trial coherence level of complex spike signals within co-activation events in alternating zones (zones 1, 3, and 5) (Figure 5J; Table S1). Zone 6 showed the same tendency. Air puffs primarily contributed to the complex spike coherence level in these zones, whereas the tone did not contribute to the coherence level (Figures 5K and 5L; Table S1). These results suggest that enhancement of sensory-evoked coherence in complex spike signals is associated with subsequent motor initiation.

To rule out the possibility that this enhancement is independent of the sensory context, we compared complex spike signals during spontaneous lick initiation outside of the task (spontaneous) with those at baseline before sensory stimuli (baseline; −2 to 0 s from the onset of sensory stimuli) (Figure S6K). Although the frequency of complex spike signals in zone 1 increased before spontaneous lick initiation (Figures S6K and S6L), their coherence level was not different between these conditions (Figure S6M; Table S1), as opposed to the significant enhancement of complex spike coherence associated with sensory-driven lick initiation (Figures 5H and 5J).

In summary, enhanced coherence of complex spike signals in alternating zones results in sensory-driven motor initiation, but not spontaneous motor initiation, suggesting a specific role of complex spike coherence in Crus I in sensory-driven motor actions when the temporal precision of the motor initiation matters.

### Coherent Complex Spike Signals in Alternating Zones Result in Temporally Precise Lick Initiation

**Figure 6:**
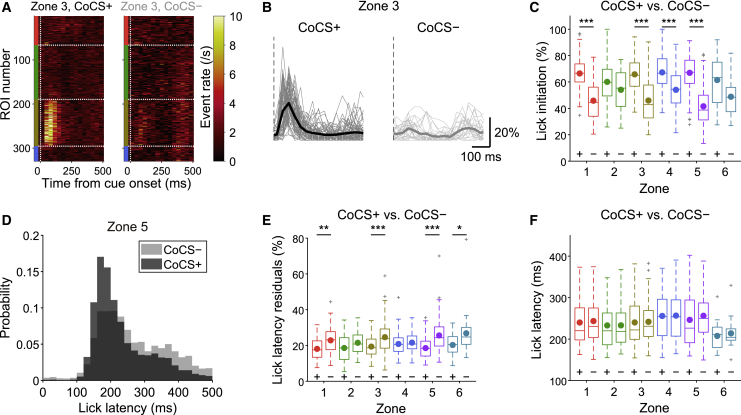
Coherent Complex Spike Signals in Alternating Zones Result in Temporally Precise Lick Initiation (A) Trial-averaged CS event rate heatmap for trials with or without co-activation (CoCS) events in zone 3 from an example session. Vertical dotted lines represent the sensory cue onset, and horizontal lines represent zonal boundaries. (B) Single-trial co-activation traces of zone 3 for trials in (A). Thin lines represent single trials, and thick lines represent trial averages. Vertical dotted lines represent the cue onset. (C) Probability of lick initiation after CoCS events (+) or no CoCS events (−) in zones 1–6 for all trials in single sessions pooled across mice. (D) Distribution of the latency of the first lick in the licking trials (Hit, FA1, and FA2 trials combined) with or without CoCS events in zone 5 pooled across animals. (E) Same as (C) but for lick latency residuals in Hit, FA1, and FA2 trials. (F) Same as (C) but for lick latency in Hit, FA1, and FA2 trials. See also Figure S7 and Table S1.

To investigate whether coherent complex spike signals in our time-demanding task influence motor initiation and its timing, we separated trials into those with (CoCS+) or without (CoCS−) coherent complex spike signals for each zone (Figures 6A and 6B, zone 3) and examined whether licking was initiated in these trials. The presence of coherent complex spike signals in the alternating zones (zones 1, 3, and 5) significantly increased the probability of lick initiation in the corresponding trials (Figure 6C; Table S1). Zone 4 showed the same trend. Moreover, among licking trials (Hit, FA1, and FA2), licks were initiated in a more precisely timed manner after coherent complex spike signals in the alternating zones (zones 1, 3, and 5) (Figures 6D and 6E; Table S1), suggesting their contribution to temporally precise lick initiation. However, lick latency remained unchanged regardless of the presence of the coherent complex spike signals in any zones (Figure 6F; Table S1). These results indicate that complex spike signals in Crus I do not influence the speed of sensory-driven motor initiation but rather contribute to its temporal precision.

### Coherent Complex Spike Signals Are Acquired Together with Precisely Timed Motor Initiation

**Figure 7:**
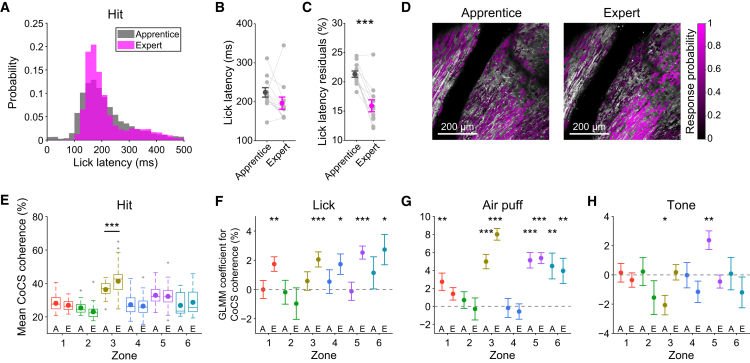
Coherent Complex Spike Signals Are Acquired, Together with Precisely Timed Motor Initiation (A) Lick latency distribution for Hit trials at early (apprentice) and late (expert) stages of task learning pooled across 11 mice. (B) Lick latency during Hit trials at early and late stages of task learning. (C) Same as (B) but for lick latency residuals. (D) ROIs colored based on CS event probability for 0–250 ms from the onset of sensory stimuli during Hit trials across early and late learning stages. (E) Comparisons of coherence level of CoCS events for zones 1–6 during Hit trials across learning. A, apprentice; E, expert. (F) GLMM coefficients for the presence of lick initiation fit on the trial-by-trial coherence level of CoCS events for zones 1–6 across learning. (G) Same as (F) but for air puff stimuli. (H) Same as (F) but for tone stimuli. See also Figure S7 and Table S1.

We next investigated whether the observed enhancement of sensory-evoked complex spike coherence and its effect on behavior change during learning. To test this, we tracked complex spike signals during learning of the multisensory association task and compared motor performance and complex spike signals across early and late stages of learning (apprentice, blocks of 20 trials; expert, randomized sequence of trials). The temporal precision of lick initiation during Hit trials improved after learning (Figure 7A). Specifically, learning resulted in more precisely timed licks (apprentice versus expert; n = 11 mice; 21% ± 1.8% versus 16% ± 3.4%; p = 7.4 × 10^−5^) (Figure 7C) without a significant change in mean lick latency (apprentice versus expert; 220 ± 40 versus 200 ± 52 ms; p = 0.15) (Figure 7B). The probability of complex spike signals during Hit trials also increased and was more focused on zone 3 after learning (Figure 7D). Furthermore, the coherence level of complex spike signals within co-activation events in zone 3 during Hit trials significantly increased after learning (apprentice versus expert; 36% ± 4% versus 41% ± 8%; p = 6.0 × 10^−4^) (Figure 7E; Table S1). To quantify the enhancement of complex spike coherence upon sensory-driven lick initiation across learning, we constructed a GLMM for early and late learning stages. The single-trial complex spike coherence level within individual zones was predicted by using the occurrence of the air puff, tone, and lick initiation. We found that a significant contribution of lick initiation to coherence level of complex spike signals emerged only after learning (Figure 7F; Table S1), whereas the contribution of the air puff mostly remained significant throughout the learning, and the tone continued to show only a minimal contribution (Figures 7G and 7H; Table S1). This indicates that the enhancement of sensory-evoked coherence in complex spike signals upon motor initiation, but not the sensory-evoked coherence itself, is acquired with learning. As for the effect of coherent complex spike signals on behavior, we found consistent effects on both probability of lick initiation and lick latency residuals early during learning (Figure S7; Table S1), as well as during all learning stages (Figure 6), indicating that the contribution of complex spike coherence to motor initiation and its timing precision remains relatively constant across learning.

Altogether, these findings suggest that the enhancement of sensory-evoked complex spike coherence in alternating zones upon motor initiation is acquired through learning, which contributes to the temporal precision of sensory-driven motor initiation.

## Discussion

We combined Neuropixels recordings of simple and complex spikes, optogenetic manipulation of simple spikes, and two-photon imaging of complex spike signals in populations of Crus I Purkinje cells during a multisensory association task to reveal that instantaneous changes in simple spike rate determine the probability and latency of motor initiation, whereas enhancement in sensory-evoked complex spike coherence contributes to its temporal precision. Our results provide a fresh perspective on two major debates in contemporary cerebellar research: (1) the contribution of simple spikes to sensory-driven motor actions, for which we show their influence on whether and when to initiate the actions, and (2) the dual roles of complex spikes in sensory processing and motor timing, for which we found them to be synergistic.

### A Multisensory Association Task Reveals a Cerebellar Role in Rapid Sensorimotor Associations

Although the cerebellum has an established role in motor coordination and plasticity (Herzfeld et al., 2018; Medina and Lisberger, 2008; Yang and Lisberger, 2014), its role in sensory data acquisition has long been debated (Bower, 1997; Gao et al., 1996; Liu et al., 2000). Here, we directly tested the role of the cerebellum in sensory-driven motor actions using a multisensory association task. The task features were specifically designed to engage the cerebellum: animals were required to (1) distinguish unisensory from multisensory information, increasing the engagement of brain regions such as the cerebellum that receive multisensory information, and (2) respond rapidly owing to the cerebellum’s role in rapid motor initiation (Rahmati et al., 2014; Welsh, 2002). Accordingly, animals learned to generate rapid and temporally precise (150–200 ms) initiation of licking, especially in response to the air puff (Figures 1D–1F).

Optogenetic manipulation of simple spike firing in Purkinje cells in lobule Crus I—one of the lobules of the lateral cerebellum—modulated sensory-driven licking (Figures 1G–1K) but did not perturb spontaneous licking (Figures S1K–S1M) or sensory-evoked licking once initiated (Figures S1N and S1O). These results provide strong support for the sensory acquisition hypothesis of cerebellar function (Bower, 1997). The suppressive effects of Purkinje cell activation on motor behavior can be explained by inhibition of cerebellar nuclei (Gao et al., 2018; Ten Brinke et al., 2017; Witter et al., 2013). Sensory-driven lick initiation during photostimulation is delayed or abolished, which provides causal evidence supporting the cerebellar contribution to motor initiation and its timing (Rahmati et al., 2014; Welsh, 2002). Altogether, our results indicate that an important role of the lateral cerebellum is to subserve rapid initiation of sensory-driven motor behavior.

### Simple Spikes Drive Sensory-Driven Motor Initiation and Modulate Its Timing

Simple spikes in Purkinje cells are sculpted by parallel fiber inputs from granule cells, which exhibit both multisensory responses (Arenz et al., 2008; Ishikawa et al., 2015) and motor-related responses (Giovannucci et al., 2017), which in turn can drive simple spike firing (Brown and Raman, 2018). However, simple spike decreases associated with learning can drive sensorimotor reflexes (Jirenhed et al., 2007). Moreover, simple spikes can show bidirectional modulation during motor behavior (Herzfeld et al., 2015, 2018; Mano and Yamamoto, 1980). Although these elegant studies have revealed the heterogeneity of simple spike responses, the contribution of simple spikes to regulating sensory-driven motor actions has remained unclear.

We used Neuropixels probes (Jun et al., 2017) to record simple spike activity simultaneously from up to 15 Purkinje cells in Crus I during our multisensory association task (Figure 2A). We found spatially intermingled Purkinje cell populations with positive and negative simple spike modulation (Figure 2G). The Purkinje cell representations of sensory modalities and motor initiation were also highly intermixed (Figure 2H), highlighting the heterogeneous nature of simple spike coding during sensory-driven behavior. Comparison with spontaneous motor initiation suggested that simple spike modulation preceding actions reflects motor initiation more strongly than sensory discrimination/perception (Figure S3).

Optogenetic stimulation of the recorded Purkinje cells resulted in bidirectional modulation in simple spike firing (Figures 3A, 3B, 3D, and 3E), with inhibitory responses presumably caused by Purkinje cell-Purkinje cell inhibitory connections (Orduz and Llano, 2007; Witter et al., 2016). The magnitude of optogenetic modulation of simple spikes was larger when lick initiation was abolished (Figure 3I), suggesting that physiological patterns of positive and negative modulation of Purkinje cell activity are critical for proper initiation of sensory-driven motor actions. Moreover, we found that optogenetic manipulation of simple spikes in both directions was correlated with a corresponding delay in lick initiation (Figures 3J–3L). We observed more positively correlated cells than negatively correlated cells: an increase in simple spikes in response to sensory stimuli delays licking in most lick-initiation-responsible Crus I Purkinje cells (Figure 3L), presumably via inhibition of downstream cerebellar nuclear neurons. Given that optogenetic manipulation did not affect the temporal variability in lick initiation (Figure 1K), it is unlikely that the delayed lick initiation was driven by precisely timed rebound firing in nuclear neurons (Person and Raman, 2011). It is also unlikely that short-latency simple spike modulation reflects motor kinematics and slowing of licking behavior, because the modulation occurred within 100 ms from the sensory cue onset, more than 50 ms before the earliest first lick, and thus could not reflect the kinematics. Therefore, we suggest that simple spikes in Crus I Purkinje cells are crucial for determining motor initiation and its timing.

### Alternating Parasagittal Organization of Sensory-Evoked Complex Spike Signals

Consistent with previous studies showing sensory-evoked complex spikes (Najafi et al., 2014a, 2014b; Ozden et al., 2009, 2012; Schultz et al., 2009; Tsutsumi et al., 2015, 2019) synchronized within zones or microzones (Ghosh et al., 2011; Mukamel et al., 2009; Ozden et al., 2009, 2012; Schultz et al., 2009; Sugihara et al., 2007; Tsutsumi et al., 2015), we found alternating parasagittal bands of sensory-responsive and non-responsive Purkinje cells (Figures 4D and 4E) (see also Romano et al., 2018). These bands were ∼300 μm wide (Figure 4K), consistent with zones rather than microzones (Andersson and Oscarsson, 1978; Ozden et al., 2009; Tsutsumi et al., 2015). Remarkably, this functional architecture is highly conserved across different animals (Figure 4J), suggesting that it plays an important role in cerebellar sensorimotor processing. Moreover, our recordings from lobule simplex showed that functionally defined zones can be assigned across lobules (Figure S5), suggesting that these zones have a similar organization, although we found some lobular specialization in response patterns (Figures S5B and S5C) (see also Heffley and Hull, 2019). Functional properties were distributed in an alternating manner, reminiscent of alternating patterns in molecular expression such as aldolase C/zebrin II expression in Purkinje cells (Sugihara and Quy, 2007; Tsutsumi et al., 2015, 2019). Although Crus I is known to be uniformly zebrin positive (Sugihara and Quy, 2007), the patterns we observed could reflect alternating expression of other molecules (Apps and Hawkes, 2009). Accordingly, whether behaviorally relevant complex spike signals observed within zebrin bands (Tsutsumi et al., 2019) can be extended across lobules represents a fruitful avenue for future work.

### Complex Spikes Represent Saliency of Sensory Information

Our results confirm recent reports that climbing fibers can respond to multisensory stimuli (Gaffield et al., 2019; Ju et al., 2019). At the behavioral level, the air puff caused quicker and temporally more precise motor initiation than the tone (Figures 1D–1F), consistent with the larger complex spike responses and stronger contribution to coherence driven by the air puff (Figure 5). Based on these findings, we speculate that the saliency of sensory inputs is represented by coherent complex spikes (Heffley et al., 2018), which could provide a platform for rapid reaction to these inputs. An alternative explanation is that complex spikes signal the value for reward prediction (Heffley and Hull, 2019; Kostadinov et al., 2019). These considerations can explain why there was no significant distinction in coherent complex spike signals between multisensory and unisensory stimuli (Figures S6A–S6F): both are perceived as sensory stimuli requiring lick initiation or strongly predicting the future reward. Therefore, multisensory integration could take place in brain regions upstream of the cerebellum, such as sensory and association cortex, which project to the inferior olivary neurons, providing climbing fiber inputs to Crus I (Shimuta et al., 2020). Simultaneous recordings from these brain regions and the cerebellum (Wagner et al., 2019) could address this possibility.

### Learned Enhancement of Coherence in Sensory-Evoked Complex Spike Signals Contributes to Temporal Precision of Motor Initiation

Given the well-established role of complex spikes in motor timing (Llinás, 2011; Welsh, 2002; Welsh et al., 1995), it is interesting to consider how Purkinje cells transform the sensory information contained in complex spike signals into temporally precise motor output. A previous study indicated that complex spikes are specifically synchronized during sensory-evoked motor actions (Welsh, 2002). We have taken these findings further by showing that complex spike coherence can be enhanced before motor initiation to contribute to sensory-driven motor initiation and its temporal precision.

We found that when the animals initiate the behavior in response to sensory stimuli, CoCS events in alternating zones in Crus I increased their coherence (Figure 5H; Figure S4E). Indeed, the presence of licking significantly contributed to single-trial coherence in complex spike signals within alternating zones (Figure 5J). A similar increase in complex spike synchrony was observed during electrophysiological recordings (Figures S4E and S4H). These observations are in line with the proposed role of synchronous complex spike signals in timed motor initiation by evoking rebound firing in downstream cerebellar nuclei (Bengtsson et al., 2011; Tang et al., 2019), i.e., the motor timing hypothesis (Jacobson et al., 2008; Llinás, 2011). In support of this idea, the presence of coherent complex spike signals significantly increased the probability of motor initiation (Figure 6C) and predicted temporally precise lick initiation in response to sensory stimuli (Figure 6E). An alternative interpretation is that this effect is driven by an increase in complex spike occurrence after the salient sensory stimuli. Our Neuropixels recordings of population complex spikes and joint peri-stimulus histogram (JPSTH) analyses suggest that there was an increase in complex spike co-occurrence (Figure S4F). However, a net increase in synchrony was also observed, and at a different timing (25 ms versus 65 ms latency) (Figure S4G). These results suggest that there could be increases in both complex spike occurrence and complex spike synchrony, which together may contribute to the enhancement of coherent complex spike signals observed in the imaging experiments. In contrast, spontaneous lick initiation was not associated with an increase in complex spike coherence (Figures S6K–S6M).

Our findings broaden the traditional view of the motor timing hypothesis in that an increase in coherence of complex spikes is particularly important in cases when a rapid response to sensory stimuli is necessary. This may occur in parallel with the recently observed ramping activity in cerebellar nuclear neurons representing motor preparation (Chabrol et al., 2019; Gao et al., 2018), which may also take place within our narrow time window (∼200 ms before lick initiation). Furthermore, we found that this enhancement of complex spike coherence upon sensory-driven motor initiation was sharpened by learning, concomitant with the improvement in timing precision of motor initiation (Figures 7C and 7F).

In summary, our results suggest that complex spike coherence can adaptively increase to ensure precisely timed motor actions driven by sensory processing, in concert with the regulation of motor latency by simple spikes. Primate and human studies have suggested that the cognitive functions of the cerebellum (Stoodley and Schmahmann, 2010) involve a combination of sensory processing (Gao et al., 1996) and central timing generation (Ivry and Keele, 1989; Jueptner et al., 1995). These functions can be reconciled by our proposed framework of cerebellar processing during rapid and precisely timed sensory-driven motor initiation, which may have implications for elucidating the cerebellar contributions to cognitive function and disease (Carta et al., 2019; Chen et al., 2014; Kelly et al., 2020; Kostadinov et al., 2019; Parker et al., 2017; Stoodley et al., 2017; Tsai et al., 2012, 2018).

## STAR★Methods

### Key Resources Table

**Table:** 

REAGENT or RESOURCE	SOURCE	IDENTIFIER
**Bacterial and Virus Strains**		

AAV1.CAG.DIO.R-CaMP2.WPRE.SV40	Penn Vector Core	N/A

**Experimental Models: Organisms/Strains**		

Mouse: C57BL/6J	The Jackson Laboratory	Stock#000664; RRID: IMSR_JAX:000664
Mouse: Pcp2(L7)-Cre	The Jackson Laboratory	Cat#010536; RRID: IMSR_JAX:010536
Mouse: Ai32	The Jackson Laboratory	Cat#012569; RRID: IMSR_JAX:012569

**Software and Algorithms**		

MATLAB	MathWorks	https://www.mathworks.com; RRID: SCR_001622
PyBehavior	Github	https://github.com/llerussell/PyBehaviour
behavior	Github	https://github.com/stsutsumi223/behavior
Suite2P	Github	https://github.com/cortex-lab/Suite2P
MLSpike	Deneux et al., 2016	https://github.com/MLspike
KiloSort2	Github	https://github.com/MouseLand/Kilosort
Phy GUI	Github	https://github.com/cortex-lab/phy
BakingTray	Github	https://github.com/SainsburyWellcomeCentre/BakingTray
StitchIt	Github	https://github.com/SainsburyWellcomeCentre/StitchIt
MaSIV	Github	https://github.com/SainsburyWellcomeCentre/masiv
Elastix	Github	https://github.com/SuperElastix/elastix
AllenCCF	Github	https://github.com/m-beau/allenCCF

### Resource Availability

#### Lead contact

Further information and requests for resources and reagents should be directed to and will be fulfilled by the Lead Contact, Michael Häusser (m.hausser@ucl.ac.uk).

#### Materials availability

This study did not generate new unique reagents.

#### Data and code availability

The codes for driving and recording the behavioral tasks supporting the current study are deposited (https://github.com/stsutsumi223/behavior). The datasets are available from the corresponding author on request.

### Experimental Model and Subject Details

#### Animals

All animal procedures were approved by the local Animal Welfare and Ethical Review Board and performed under license from the UK Home Office in accordance with the Animals (Scientific Procedures) Act 1986. Two-photon imaging experiments were performed in Pcp2(L7)-Cre mice (n = 4), maintained in-house by crossing to C57/BL6J wild-type mice. Optogenetics experiments were performed in Pcp2(L7)-Ai32 mice (Pcp2(L7)-Cre mice crossed with Ai32 reporter line; n = 4), and in C57/BL6J wild-type mice (n = 3 mice). Neuropixels recordings with optogenetics were performed in a separate cohort of Pcp2(L7)-Ai32 mice (n = 4). Age of mice was over P60 for all experiments. Both female and male mice were used for the study. All mice were house in an enriched environment within a temperature- and humidity-controlled, specific-pathogen free barrier facility at UCL. Female mice were group housed and male mice were single housed. All mice were maintained on a 12:12 day-night cycle.

### Method Details

#### Surgery

At least 40 min before surgery, mice were intraperitoneally injected with a cocktail of dexamethasone (Dexadron, 5 mg/kg) and buprenorphine (Vetergesic, 1 mg/kg) to reduce brain swelling and pain during surgery. Mice were then anesthetized with isoflurane (5% induction and 1%–2% maintenance) throughout the surgical procedure. Pedal pinch-reflex was used to monitor the depth of anesthesia. A custom-made head plate with a circular inner opening of 7 mm diameter was fixed over the left cerebellar folium Crus I (1.5 mm caudal and 3.5 mm lateral from lambda) and secured with dental cement (Super-Bond C&B, Sun-Medical). A 3 mm craniotomy, centered in the middle of the head plate hole, was then performed to expose the cerebellar cortex for virus injection and window installation. The dura was kept intact. For imaging experiments, a Cre-dependent R-CaMP2 virus (AAV1.CAG.DIO.R-CaMP2.WPRE.SV40) diluted at 1:5 from stock titer was injected at 3 locations to cover the entire Crus I. At each location, 400 nL of virus solution was pressure-injected at depths of 100–150 μm below the cerebellar surface: the virus-containing pipette was tilted by 35° from vertical line, and injections were performed at 160–200 μm axial depth in 10 μm steps, at 80 nL/min for total 5 min. We waited 5 min after each set of injections before retracting the injection pipette from each location to avoid viral reflux. In total, 1.2 μL of diluted virus was injected per mouse. No virus was injected for optogenetics experiments. Finally, a 3 mm single-paned coverslip was press-fit in to the craniotomy, sealed to the skull by a thin layer of cyanoacrylate (VetBond) and fixed in place by dental cement. For optogenetic experiments, black pigment was added in the dental cement to minimize scattering of LED light for photostimulation. All non-Crus I regions were covered with a layer of black dental cement to minimize the possibility of stimulating these regions while ensuring Crus I stimulation. The conical portion of a nitrile rubber seal (RS Components, Stock no. 749-581) was then glued to the head plate with dental cement to prevent mice from directly seeing the LED light for photo-stimulation. The window was filled with Kwik-Cast to protect the window preparation during recovery and between recording sessions. Mice were allowed to recover for a minimum of 7 days and given post-operative analgesia as needed.

#### Task training

After mice had recovered from surgery, they were placed under water restriction for 1–2 days when they were acclimated to the recording setup. All mice were maintained at 85%–88% of their initial body weight over the course of recording experiments. Water was provided only from the lick port during task training. In cases mice failed to maintain the body weight, additional water (1–2 mL) was provided after the task. Mice were trained once daily. Mice typically underwent 2–3 days of habituation sessions where every lick on a lick port was rewarded with 2 μL of a saccharine solution (1.4 mg/L) at a maximum rate of 2 μL/s. Once mice were well-habituated to the setup and reliably started licking to the lick port, a multisensory association task was initiated. We presented two modalities of sensory cues to the animals: an air puff to the center of the left whisker pad generated by a Picospritzer at 5–6 psi and/or auditory tone stimulation (3.3 kHz, 80 dB). When the mice licked during the simultaneous presentation of these two sensory stimuli for 0.5 s (Go, Figure 1B), they were rewarded with 2 μL of a saccharine solution (Hit). On the other hand, when the mice licked during the 0.5 s presentation of either air puff alone (No-Go1) or tone alone (No-Go2), they were punished with white noise for 0.5 s (false alarm (FA) 1 and 2). Neither no licking to either of the single sensory cue (correct rejection (CR) 1 and 2), nor to the multisensory cue (Miss) were rewarded or punished. Ratio of each trial type was calculated per stimulus condition (e.g., CR1 rate = N(CR1) / (N(CR1) + N(FA1))). Mice had to withhold licking for a randomized interval (1 ± 0.5 s) before the next cue, to prevent them from continuous or predictive licking. Inter-trial interval was set to 3.5 s. We started with alternating presentations of blocks of 20 trials (20 Go trials – 20 No-Go1 trials – 20 Go trials – 20 No-Go2 trials) to facilitate mice to learn the contingencies. For the initial 1–2 training sessions, the reward was automatically given during the Go cue at 0.2 s latency for the mice to associate combined sensory stimuli with water reward. As soon as mice began to associate the multisensory stimuli with licking, we turned off the auto-reward (early learning stage). When the CR rate for both sensory stimuli exceeded 80% in a block trial session, we pseudo-randomized the trial sequence at a ratio of 1:1:1 Go/No-Go1/No-Go2 trials, with a maximum of three consecutive trials of the same cue. To facilitate the rejection of licking to single sensory stimuli, we repeated the same cue for three trials maximum if the decision was incorrect (repeat-if-incorrect), until the CR rate for both sensory stimuli exceeded 80%. Finally we turned off the repeat-if-incorrect configuration (late learning stage). Performance of mice was determined by sensitivity index (d’) (Huber et al., 2012) calculated separately for No-Go1 and No-Go2 trials, and the minimum of these values was taken into account. d’ value was given by Z(Hit rate) − Z(FA rate) based on an inverse of cumulative distribution function of a normalized distribution whose mean is 0 and standard deviation is 1 (e.g., Hit rate = 97.5% roughly corresponds to a z score of 2, and FA rate = 2.5% roughly corresponds to a z score of −2, thereby d’ = 2 − (−2) = 4) (Macmillian and Creelman, 2004). This metric represents how well mice discriminate Go cue and No-Go cue. If Hit rate was 100% in an imaging session, we assigned Hit rate as 99% to avoid the d’ to be infinite. Mice were trained until the minimum d’ for a randomized trial sequence exceeded 2.5. Of the total 11 mice trained, 9 mice reached the expert performance. We switched trial sequences either within a session or across sessions (days). The task was coordinated by PyBehavior (https://github.com/llerussell/PyBehaviour) and custom-modified codes (https://github.com/stsutsumi223/behavior). Task events were recorded at 3,000 samples/s using a MATLAB code (https://github.com/stsutsumi223/behavior). For tone-attenuated sessions (Figures S6G–S6J), each tone level (58, 63, and 67 dB) was randomly interleaved in 10% of all tone-containing trials.

#### Optogenetics experiments

Pcp2(L7)-Ai32 (n = 4 mice) and wild-type mice (n = 3 mice) were first trained until they achieved the performance level of d’ > 2 using randomized sequence trials of the multisensory association task for at least 2 days. In each session, these mice underwent 300 trials of the randomized trial sequence (Go:No-Go1:No-Go2 = 1:1:1) where in one third of Go trials (32.8 ± 0.2 trials) they received photostimulation using a 470 nm LED coupled with an optical fiber (M470F1, Thorlabs) directed to the cranial window. Because strong photostimulation caused complete cessation of licking during Go trials, power was titrated for individual mice to cause 21%–85% reduction of Hit rate (the power used was 0.5–6.0 mW). Light powers were calibrated daily by either a photodiode power sensor (S130C, Thorlabs) or a digital optical power meter (PM100D, Thorlabs). The duration of photostimulation in each trial corresponded to the length of the sensory stimulus (500 ms). In control experiments, trained mice were allowed to obtain a saccharine solution every time they spontaneously licked to the lick port (max 2 μL/s, 500 ms response window and 300 ms inter-trial interval, without withhold periods). They typically continued licking for several minutes, during when LED photostimulation was delivered at one third of these 500 ms response windows (Figures S1K–S1M). As an external control, wild-type mice received the same training protocol and the same LED illumination during Go trials as Pcp2-Ai32 mice (Figures S1F–S1J).

#### Neuropixels recordings

Pcp2(L7)-Ai32 mice (n = 4) were first trained until they achieved the performance level of d’ > 2 with randomized sequence trials of the multisensory association task for at least 2 days. LED illumination power was titrated as stated above. On the recording day, a small hole (∼1 mm diameter) was drilled on the coverslip and the dura was carefully removed under isoflurane anesthesia (5% induction and 1%–2% maintenance). The drill hole was protected with Kwik-Sil. After this procedure, the mice were placed in the home cage for > 30 min for recovery. Right before the recording, the Neuropixels probes were manually coated with lipophilic dyes (DiI or DiD). The probes were fixed to a custom-made 3D printed holder and handled using a Sutter micromanipulator. The probe was lowered in the coronal plane at 37 degrees from horizontal at 4 μm/s until 1500–2000 μm below pia. They were left to settle for about 15 min to reduce subsequent drift due to brain relaxation post-insertion. Recording was started before and stopped after behavioral sessions. Recordings were obtained at 30,000 samples/s. In each session, the mice underwent a randomized trial sequence (Go:No-Go1:No-Go2 = 1:1:1). During 33% of Go trials, they received photostimulation. After the recording finished, the probe was carefully retracted and soaked overnight in Tergazyme.

#### Neuropixels analysis

Spike sorting was carried out using KiloSort2 (https://github.com/MouseLand/Kilosort). Sorted clusters were manually curated with Phy template GUI (https://github.com/cortex-lab/phy). Simple spike clusters were identified on the basis of their location in a Purkinje cell layer (Figure S2A), waveform (Figures S2B and S2C), regularity (denoted by the oscillatory fashion of their auto-correlogram; Figure S2D), and firing rate (> 40 spikes/s; Figure S2H). Simple spike and complex spike clusters arising from the same Purkinje cells were identified by cross-correlating the clusters of simple and complex spike, using the characteristic simple spike pause following the complex spike signature (Kostadinov et al., 2019) (Figure S2G). In some cases, we identified the clusters with a firing rate < 40 spikes/s and > 25 spikes/s as simple spike clusters when they showed a pause in the cross-correlogram with a complex spike cluster, or when they were located in the middle of a group of Purkinje cells, and the waveform and the auto-correlogram were similar to those of the other neighboring simple spike clusters. Complex spike clusters were determined on the basis of their location in a Purkinje cell layer (Figure S2A), characteristic waveform (Figures S2B and S2E), regularity (Figure S2F), the existence of a pausing cross-correlogram with a simple spike cluster (Figure S2G), and their mean firing rate (∼1.5 spikes/s; Figure S2H). Spike times were down-sampled at 3,000 samples/s to align with the task events. For complex spike synchrony at 1 ms bins (Figure S4E), we extracted significantly synchronized events within 0–50 ms window from the onset of sensory stimuli as > 97.5 percentile of peak synchrony level calculated from 1,000 times time-shuffled spikes at the same window for each trial, and compared the level of synchrony within these events. Joint peri-stimulus histogram (JPSTH; Figures S4F–S4H) of complex spike pairs at 5 ms bins was calculated as in the literature (Schultz et al., 2009; Welsh, 2002) and averaged across all pairs within Crus I.

#### Histology

Under deep ketamine-xylazine anesthesia, mice were transcardially perfused with 4% paraformaldehyde in 0.1 M sodium phosphate buffer and post-fixed overnight. After fixation, we embedded the brains in 5% oxidized agarose (Type-I agarose, Merck KGaA, Germany) and covalently cross-linked the brain to the agarose by incubating overnight at 4°C in 0.5%–1% sodium borohydride (NaBH_4_, Merck KGaA, Germany) in 0.05 M sodium borate buffer. We imaged the brains in a custom-made serial two-photon tomography microscope (Mayrhofer et al., 2019), controlled using a MATLAB-based software (ScanImage 2017b, Vidrio Technologies, USA) and BakingTray (https://github.com/SainsburyWellcomeCentre/BakingTray, extension for serial sectioning). The setup consists of a two-photon microscope coupled with a vibratome (VT1000S, Leica, Germany) and a high-precision X/Y/Z stage (X/Y: V-580; Z: L-310, Physik Instrumente, Germany). The thickness of a physical slice was set to be 50 μm for the entire brain and we acquired optical sections at 5 μm using a high-precision piezo objective scanner (PIFOC P-725, Physik Instrumente, Germany) in two channels (green channel: 500–550 nm, ET525/50, Chroma, USA; red channel: 580–630 nm, ET605/70, Chroma, USA). Each section was imaged by 7% overlapping 1025 × 1025 μm tiles. A 16X water immersion objective lens (LWD 16X/0.80 NA; MRP07220, Nikon, Japan), with a resolution of 0.8 μm in X and Y and measured axial point spread function of ∼5 μm full width at half maximum. After image acquisition, the raw images were stitched using a MATLAB-based software (StitchIt, https://github.com/SainsburyWellcomeCentre/StitchIt). This software applies illumination correction on the basis of the average tile in each channel and optical plane and subsequently stitches the tiles from the entire brain. After stitching and before further image processing, we down-sampled the stitched images by a factor of 6 in X and Y obtaining a voxel size of 25 × 25 × 25 μm, using a MATLAB-based software (MaSIV, https://github.com/SainsburyWellcomeCentre/masiv). The auto-fluorescence channel of this 3D stack was then registered to the Allen brain 25 × 25 × 25 μm template using Elastix (https://github.com/SuperElastix/elastix) command line tool, with consecutive affine and B-spline transformations. The resulting transformation parameters were then applied to the channel containing the electrode track, which was 3D registered to the Allen atlas and fed to a custom-made Allen-CCF alignment tool (MATLAB, https://github.com/m-beau/allenCCF, modified from Shamash et al., 2018). This allowed us to precisely determine in which cerebellar lobule each recording channel was located (Figure S2A).

#### Behavioral analysis

Lick latency was calculated as the time between cue onset and first lick within a trial. Onset of spontaneous licking was extracted as the first lick in a trial which was outside of the sensory cues, therefore after CR1, CR2, or Miss trials (Figure S6; Spontaneous). For the spontaneous licking in control experiments, lick latency was calculated based on the time between an arbitrary trial onset and the first lick within a trial (Figure S1K). Lick latency residuals were defined as the absolute difference between the lick latency in a given trial and the median of lick latency of the same trial type within the session. These values were normalized by the mean lick latency and shown in percentage. Ratio of licking epochs was calculated as the ratio of 500 ms arbitrary response windows containing at least one lick (Figure S1M).

#### Two-photon imaging

During the multisensory association task, two-photon imaging was performed using a custom-built two-photon microscope (Sutter Instruments) equipped with a 16X/0.8 NA objective lens (Nikon). The microscope was controlled using ScanImage software (Vibrio Technologies) in conjunction with MATLAB (R2015b, MathWorks). Two-photon excitation was achieved with a pulsed Ti:Sapphire laser (Mai Tai HP; Spectra-Physics) at a wavelength of 980 nm. Laser power on sample was adjusted to be less than 40 mW. Fluorescence signals were divided into green and red channels with a dichroic mirror and emission filters (Chroma) and detected with GaAsP photomultiplier tubes (Hamamatsu Photonics). A 660 × 660 μm field of view (Figure 4A) was scanned using a resonant scanning galvanometer (8 kHz, Cambridge Technologies) at a resolution of 512 × 512 pixels. 15,400 frames were acquired at 30.03 frames/s (513 s) per imaging session. The small delay in scanning between top and bottom parts of the imaging field was not taken into account for subsequent analyses. Imaging was focused on the left Crus I and at least one imaging video was acquired per field of view per session (day).

#### Imaging analysis

Imaging data were analyzed using MATLAB software (R2019a, MathWorks). To correct motion artifacts in the x-y plane and extract regions of interest (ROIs) corresponding to PC dendrites, we used Suite2P (Pachitariu et al., 2017). Parameters used for ROI extraction were: Nk0 = 1,300 (number of clusters to start with), Nk = 650 (number of clusters to end with), sig = 0.5 (spatial smoothing length in pixels) and diameter = 2 (expected diameter of cells). After image registration and automatic extraction of ROIs, ROIs corresponding to PC dendrites were manually selected and non-PC dendrite-like ROIs were curated using the following criteria: 1. ROI shape was apparently not like a PC dendrite, such as small, round or vessel-shaped, 2. the fluorescence trace generated by mean fluorescence of pixels in the ROI did not show characteristic fluorescence increases caused by climbing fiber inputs (fast rise and slow decay), 3. calcium signals were saturated, showing sustained fluorescence increases on the order of seconds, 4. signal-to-noise ratio was too low. To reduce multiple counts of single PC dendrites as a result of oversegmentation, correlation coefficients between calcium traces were plotted against the distances between ROIs, and both distance and correlation thresholds for merging ROIs were selected individually for each imaging session based on the scatterplot. The range of thresholds was 5–20 μm for distance and 0.5–0.75 for correlation coefficients. This process was iterated until no ROI pairs satisfied these criteria. The result of merging was visually inspected and the merging was restarted if ROIs were either under- or over-merged. We extracted 297 ± 15 dendritic regions of interest (ROIs) per field of view (mean ± SD, n = total 12 fields of view in 4 mice; Figures 4A, 5C, and 5E). A weighted average of the fluorescence trace of each group of merged dendritic segments was computed based on the number of pixels in each segment. Correlation matrix was calculated based on the correlation coefficient between individual fluorescence traces from ROIs aligned from lateral to medial, during a whole imaging session (Figure 4F).

#### Event detection and synchronization

ΔF/F was calculated from raw fluorescence traces using the following equation: (F – F_0_) / (F_0_ – F_b_), where F is a raw fluorescence value, F_0_ is an 8th percentile of the fluorescence values surrounding 1 s (–15 to +15 frames from each frame, total 31 frames) and F_b_ is a minimum fluorescence value of the mean image. An event detection algorithm, MLspike (Deneux et al., 2016), was used to identify fast dendritic calcium transients, faithful indicators of complex spikes in Purkinje cells, in each dendritic ROI. As an input to MLspike, we used ΔF plus the maximum value of each ΔF trace (ΔF_max_). The baseline fluorescence parameter (F_0_) was set as ΔF_max_, the sampling rate (dt) was set at 1/30.03, and the indicator decay parameter (tau) was set to 0.15. The estimated height of single spikes (a) was set to 0.65. The output of MLspike is an event time, as well as an amplitude (an integer multiple of the unitary event size detected of each trace). Events detected in consecutive bins, which are very likely to reflect a large dendritic event corresponding to a single complex spike rather than multiple separate complex spikes at our imaging rate (30.03 frames/s), were summed and binned at the first time point of each sequence. We binarized these events because amplitudes of more than 1 rarely occurred thanks to the relatively linear relationships between fluorescent signals and the number of spikes for R-CaMP2 (Inoue et al., 2015). All behavioral parameters together with two-photon imaging frame acquisition times were acquired simultaneously and digitized at 3,000 samples/s using a National Instruments data acquisition board (NI USB-6351) and saved using a custom-modified MATLAB code (softwareAnalogTriggerCapture). Subsequent analysis was performed offline using custom-written MATLAB codes (R2019a). Imaging frames (30.03 frames/s) were aligned to task events (3,000 samples/s) by using frame trigger signals (3,000 samples/s). For heatmaps (Figure 4, Figure 5, Figure 6 and Figures S5–S6), the timing of task events were assigned as a half of the frame length (0.5/30.03 s) before the first imaging frame triggered after the task events. Response probability was quantified as the probability of trials with 1 or more of the extracted complex spike events within the 250 ms window (Figure 4E).

#### Extraction of zones and CoCS events

To extract spatial and functional clusters of population complex spikes (zones), semi-automated sequential k-means clustering (Ozden et al., 2008) on correlation matrices was performed for individual imaging data. K-means clustering with cluster number of two was sequentially performed until there were no separable components within each cluster (i.e., further separation of the cluster causes random separation of ROIs within the cluster). ROIs within each zone were spatially aligned from lateral to medial, then the zones were spatially aligned from lateral to medial (Figure 5A). Absolute zonal coordinates were: 4.15 ± 0.04, 3.93 ± 0.04, 3.79 ± 0.03, 3.59 ± 0.06, 3.24 ± 0.11, 2.94 ± 0.08 mm lateral from lambda for zones 1–6 (mean ± SD, n = 4 mice), assuming that center of middle fields of view corresponds to the center of the craniotomy (3.5 mm lateral from lambda).

To extract coherent complex spike signals from individual zones, the co-activation (Ozden et al., 2012) trace, the fraction of ROIs co-activated in an imaging frame, was first obtained (colored traces in Figure 4G). Co-activation (CoCS) events were then defined as a local peak in the co-activation trace exceeding a z-score of 3 (black dots in Figure 4G). Coherence level was defined as the peak value of each CoCS event, which corresponds to the proportion of co-activated dendrites within individual zones at 33 ms (1,000/30.03 ms) bins. CoCS events were aligned to the task events, and probability for individual zones was calculated using the same 250 ms window as the spike events (Figures 4 and 5 and Figure S6). For baseline and spontaneous licking, CoCS events at –250 to 0 ms from cue onset or lick onset were used for calculating coherence level for individual zones (Figure S6).

#### Generalized linear model (GLM)

For GLM analyses on Figures 2 and S3, we fit a single trial simple spike rate modulation (0–100 ms from cue onset compared with for Figure 2 and –100 to 0 ms from lick onset for Figure S3) in single Crus I Purkinje cells by using the presence or absence (1 or 0) of the air puff, tone, and licking in the trial, as in the following equation:

Simplespikemodulation=β0+β1×(air puff)+β2×(tone)+β3×(lick)

where β is coefficient of the fit, minimizing the difference between the model and actual data. We used MATLAB function fitglm to perform this calculation. We assumed normal distribution for the simple spike modulation. Significance of coefficient for each fixed effect was determined by comparing model fit by removing that variable from the model (leave-one-out procedure). Fitness of the model was assessed with coefficient for determination (R^2^; Table S1). For Figure S3, in order to include non-licking trials, which is necessary for using the lick predictor in our model fitting procedure, we aligned the simple spike rate of non-licking trials (CR1, CR2, and Miss) to the average lick latency in the corresponding licking trials (FA1, FA2, and Hit).

#### Generalized linear model with mixed effects (GLMM)

For GLMM analyses in Figures 6 and 7, we fit a single trial coherence of CoCS events in single zones by using the presence or absence (1 or 0) of the air puff, tone, and licking in the trial as fixed effects and a mouse label as a random effect, as in the following equation:

Coherencelevel=β0+β1×(air puff)+β2×(tone)+β3×(lick)+b

where β is coefficient of the fit, minimizing the difference between the model and actual data, and b is a random effect, considering across mouse variance. We used the MATLAB function fitglme to perform this calculation. We assumed normal distribution for the coherence level. Significance of coefficient for each fixed effect was determined by comparing model fit by removing that variable from the model (leave-one-out procedure). Fitness of the model was assessed with the coefficient for determination (R^2^; Table S1).

### Quantification and Statistical Analysis

All statistical analyses were performed using a MATLAB software (R2019a, MathWorks). All statistical tests were two-tailed, and significance was assigned at p < 0.05. Data presented in the text are mean ± SD and those in the figures are mean ± SEM; ns: p ≧ 0.05; ^∗^p < 0.05; ^∗∗^p < 0.01; ^∗∗∗^p < 0.001 unless otherwise stated. For multiple comparisons, we corrected p values by using either the Bonferroni method, Tukey’s method, or the Benjamini-Hochberg method (Benjamini and Hochberg, 1995). For analyses in Figures 7 and S7, only the imaging sessions with at least 7 trials each with or without CoCS events during licking trials were taken into account. All the statistical tests and results are shown in Table S1.

## Supplemental Information

Document S1. Figures S1–S7

Table S1. A Table Presenting All Statistical Results, Related to Figures 1, 2, 3, 6, 7, S1, S3, S4, S6, and S7 Complete table of all the statistics.

Document S2. Article plus Supplemental Information
