# Supplementary material for: Purkinje Cell Activity Determines the Timing of Sensory-Evoked Motor Initiation
Source: Cell Rep. 2020 Dec 22;33(12):108537. doi: 10.1016/j.celrep.2020.108537 (PMC7773552; doi:10.1016/j.celrep.2020.108537)
Supplement: Document S1. Figures S1–S7 [file mmc1.pdf]

Cell Reports, Volume *33*

Supplemental Information

Purkinje Cell Activity Determines the Timing of Sensory-Evoked Motor Initiation

Shinichiro Tsutsumi, Oscar Chadney, Tin-Long Yiu, Edgar Bäumler, Lavinia Faraggiana, Maxime Beau, and Michael Häusser

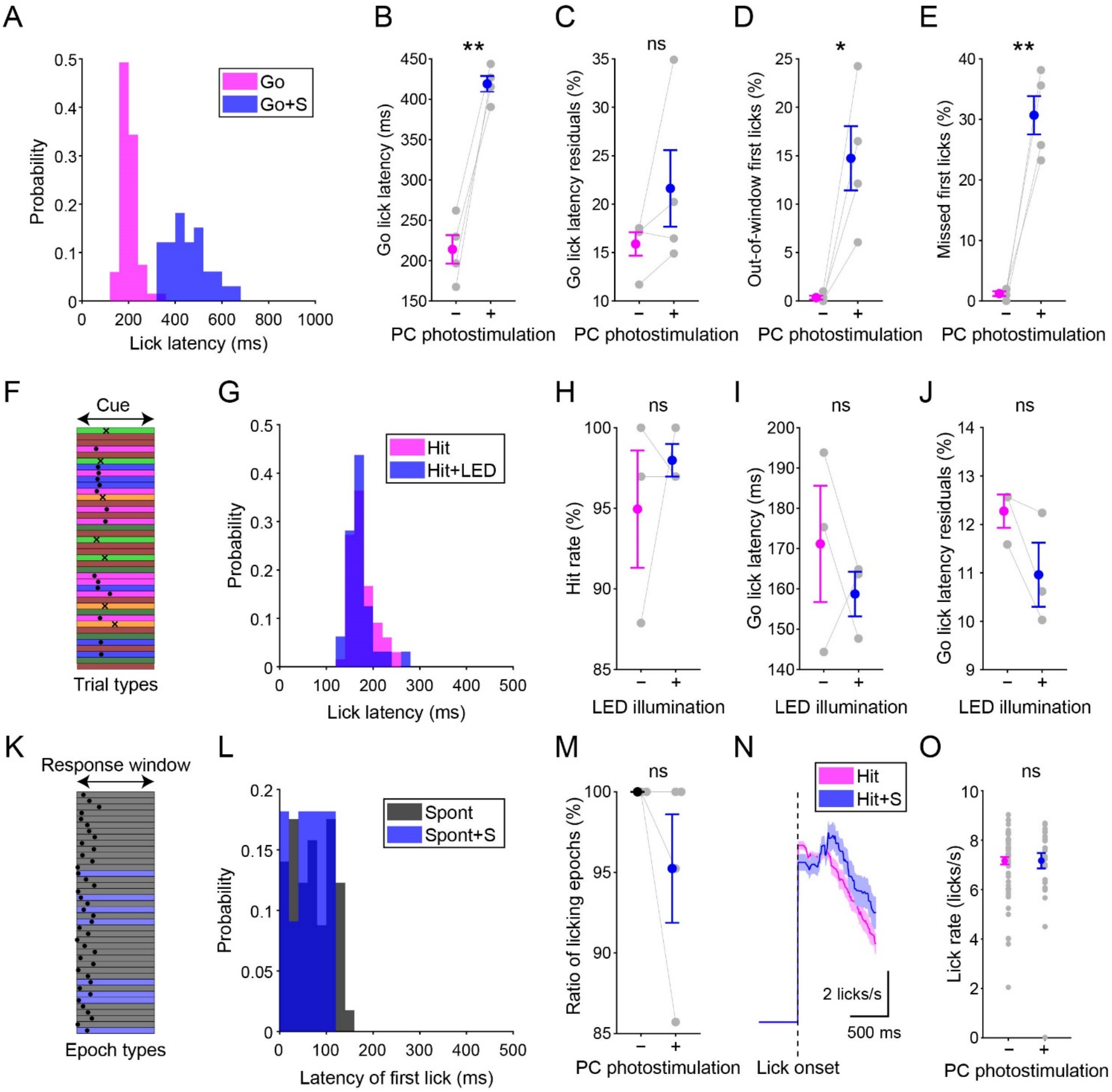

Figure S1. PC photostimulation can either delay or abolish motor initiation and specifically disrupts sensory-driven behavior. Related to Figure 1

(A) Lick latency distribution after the onset of Go cues in the presence (Go+S) or absence (Go) of LED photostimulation in a representative session (n = 67 and 33 trials for Go and Go+S). (B) Lick latency after the onset of Go cue in the absence (−) or presence (+) of LED photostimulation (N = 4 mice). (C) Same as (B), but for lick latency residuals. (D) Same as (B), but for the ratio of out-of-window (500–1000 ms after the onset of Go cue) first licks. (E) Same as (B), but for the ratio of missed first licks. (F) Representative performance of a wild type mouse in a single session with LED illumination. Colors represent trial types. Dots represent first licks for Hit trials. Crosses represent first licks for FA trials. (G) Lick latency distribution for Hit trials in the presence (Hit+S, blue) and absence (Hit, magenta) of LED illumination from a representative animal (n = 32 and 66 trials from single mouse). (H) Hit rate in the absence (−) or presence (+) of LED illumination in wild type mice (N = 3 mice). (I) Same as (C) but for lick latency in Hit trials. (J) Same as (C) but for lick latency residuals in Hit trials. (K) Representative performance during spontaneous licking with or without LED photostimulation (Spont+S and Spont, respectively). Colors represent epoch types (Spont, gray and Spont+S, blue). Dots represent first licks in the pre-specified 500 ms epochs. (L) Lick latency distribution during spontaneous licking in the presence

(Spont+S) or absence (Spont) of LED photostimulation from a representative animal (n = 11 and 57 trials from single mouse). (M) Ratio of licking epochs in the absence (−) or presence (+) of LED photostimulation in Pcp2-Ai32 mice (N = 4 mice). (N) Trial-averaged and lick onset-aligned lick rate for Hit trials in the presence (Hit+S) or absence (Hit) of photostimulation for a single session in a representative animal (n = 30 and 72 trials). Lick rate is shown in mean ± S.E.M. (O) Comparison of lick rate within 500 ms from the lick onset in the absence (−) or presence (+) of photostimulation (n = 72 and 30 trials). (B–E, H–J, and M) Gray dots and lines represent mean for individual mice. ns: $p \geqq 0.05$; *$p < 0.05$; **$p < 0.01$; paired t-test. (O) Gray dots represent individual trials. ns: $p \geqq 0.05$; Mann-Whitney U test. See also Table S1.

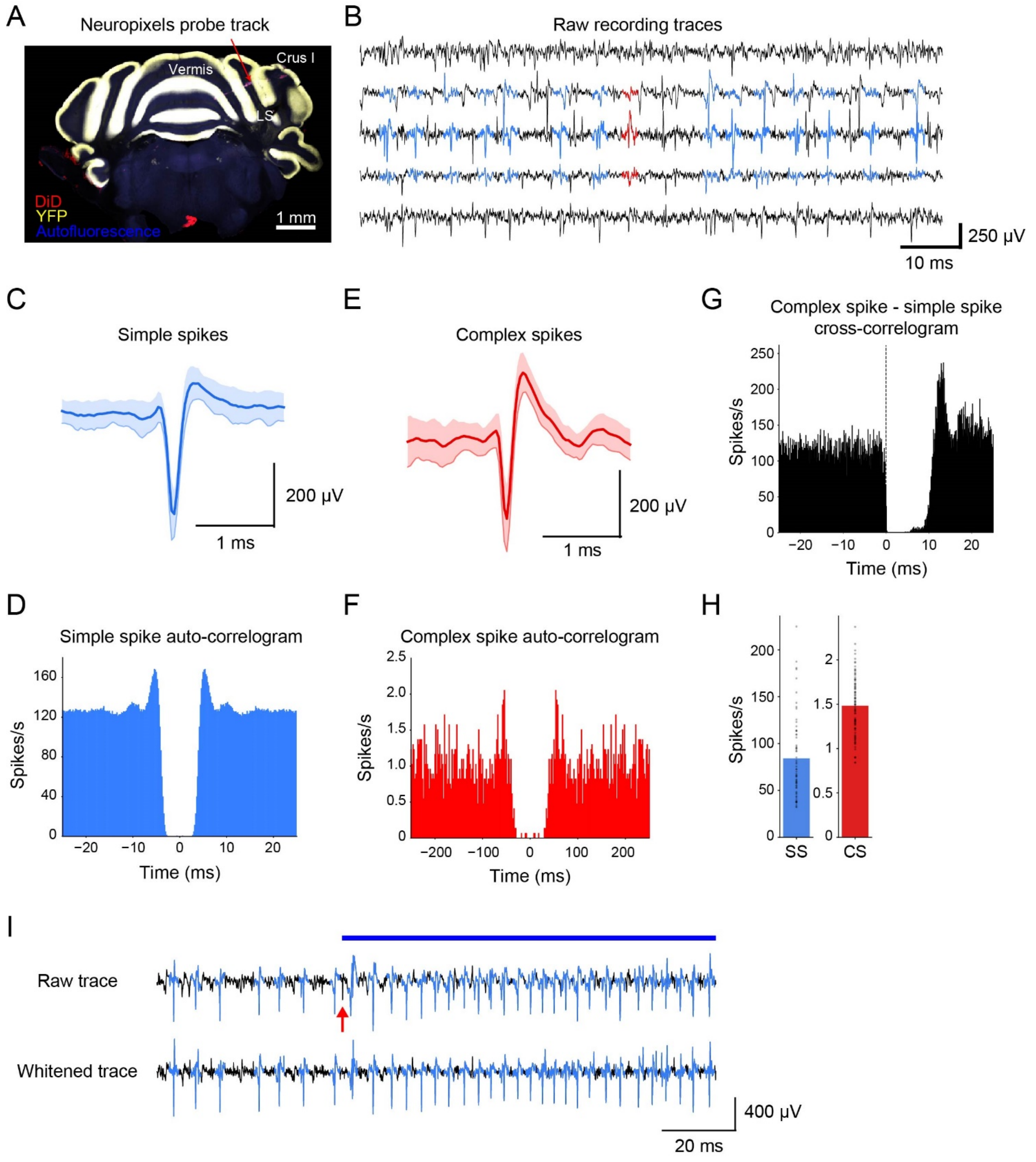

Figure S2. Neuropixels recordings of simple and complex spikes. Related to **Figures 2 and 3**

(A) Maximum projection image of a brain slice in a Pcp2-Ai32 mouse recorded with a Neuropixels probe. Red color represents the probe track (DiD staining). Yellow color represents YFP expression in Purkinje cells. Autofluorescence is in blue. Red arrow indicates the location of the Purkinje cell clusters described in (C–G). Cerebellar lobules are indicated in white letters. LS, lobule simplex. (B) Representative raw traces recorded from 5 consecutive channels on a Neuropixels probe (20 µm separation, located at the arrowhead in (A)). The colors highlight 3 ms snippets around spike times of representative simple spikes (blue) and complex spikes (red) originating from the same Purkinje cell. (C) Mean waveform of the simple spikes in (B) at the peak channel. The thick line and shaded area represent mean ± S.D. (n = 100 spikes spanning a 1-hour recording). (D) Auto-

correlogram of simple spikes in (C) (0.2 ms bins, 60 ms window). (E) Same as (C), but for the complex spikes in (B). (F) Same as (D), but for the complex spikes in (B) (2 ms bins, 600 ms window). (G) Cross-correlogram between simple and complex spikes in (B). The characteristic pause in simple spiking after complex spiking confirms that these spikes originate from the same Purkinje cell. (H) Mean firing rate for all recorded simple spikes (SS, blue; N = 4 mice; 64 units) and complex spikes (CS, red; N = 4 mice; 87 units) across lobules. Individual dots represent single units. (I) An example raw trace (top) and whitened trace (bottom) from a Neuropixels recording during an epoch of photostimulation indicated by a blue horizontal line. Light blue waves indicate simple spikes. Note the increase in simple spike rate during photostimulation, and absence of photo-artifact (red arrow) in a whitened trace.

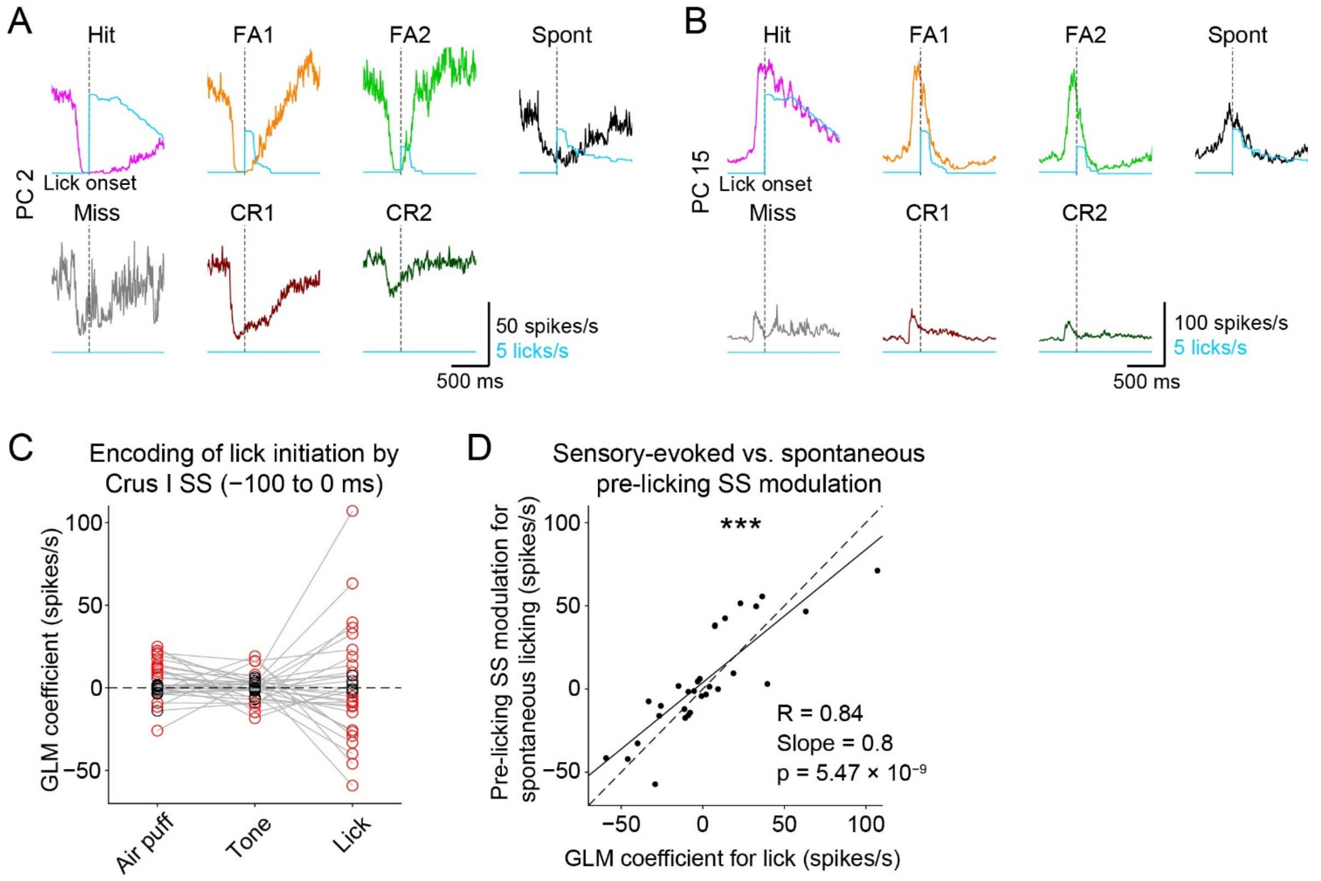

Figure S3. Pre-licking representation in Crus I simple spikes is consistent across context. Related to **Figure 2**

(A) Trial-averaged simple spike (SS) firing rate aligned to the lick bout onset from a representative Purkinje cell (PC 2 in Figure 2) overlaid by the trial-averaged lick rate for each trial type. (B) Same as (A) but for PC15 in Figure 2. (C) GLM coefficients for the air puff, tone, and lick initiation fit to SS modulations at −100 to 0 ms from the onset of lick bout onset in individual Crus I Purkinje cells (N = 4 mice; 30 cells). Gray lines represent individual cells. Red circles represent significant ($p < 0.05$) contributions of the predictors to the model and black circles represent non-significant ones; GLM leave-one-out coefficient test. (D) Linear regression of GLM coefficient for lick in (C) to spontaneous SS modulation for all recorded Crus I PCs (N = 4 mice; 30 cells). The dotted line represents the unity line and the solid line represents the fitted line. ***$p < 0.001$; ANOVA on linear regression. See also Table S1.

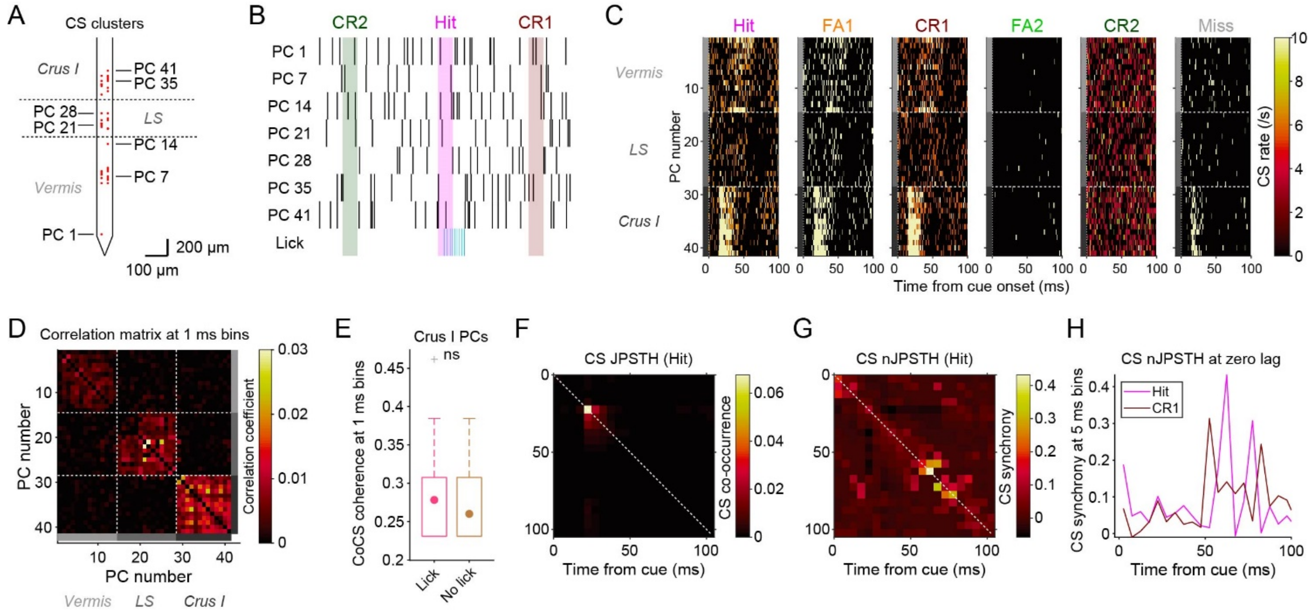

Figure S4. Neuropixels recordings of complex spikes are consistent with imaging results. Related to Figures 4 and 5

(A) Positions of complex spike (CS) clusters on a Neuropixels probe. Red dots represent the positions of channels where each cluster was recorded. Dotted lines represent the positions of the boundaries between cerebellar lobules which are color-coded. PC, Purkinje cell; LS, lobule simplex. (B) Representative raster plots of CS and licks during the task. Colored shadings represent the duration of sensory stimuli (500 ms) for the corresponding trial type. (C) Heat maps for trial type-averaged firing rate of CS within 1 ms bins. Purkinje cells (PCs) are aligned on the basis of spatial positions of the recorded channels. Colored bars on the left represent color-coded cerebellar lobules as in (A). Note the strong responses almost exclusively in Crus I. (D) Spontaneous correlation matrix of CSs at 1 ms bins calculated from spike rasters at −2 to 0 s from the onset of sensory stimuli. PCs are aligned on the basis of spatial positions of the recorded channels. Colored bars represent color-coded cerebellar lobules as in (A). Note the CS synchrony within each lobule but not across lobules. (E) The level of coherence within co-activated complex spikes (CoCS) at 1 ms bins in Crus I during licking and non-licking trials with air puff (Hit+FA1 and Miss+CR1, respectively) from a representative animal (n = 13 PCs). Edges of the boxes are interquartile range, and dots represent mean (ns: $p = 0.11$, paired t-test). (F) Trial and population-averaged joint peri-stimulus histogram (JPSTH) of CSs at 5 ms bins during Hit trials between all Crus I PC pairs (n = 13 PCs, total 78 pairs) in a representative animal. A dotted diagonal line represents zero lag CS co-occurrence between PC pairs. Note the distinct peak in CS co-occurrence at 20–25 ms bin from the onset of sensory stimuli. (G) Same as (F), but normalized by standard deviations for each time bin. A dotted diagonal line represents zero lag CS synchrony between PC pairs. Note the peaks in synchrony around 60–80 ms from the onset of sensory stimuli. nJPSTH, normalized JPSTH. (H) Zero lag synchrony between PC pairs (the diagonal line in (G)) during Hit (magenta) and CR1 (purple) trials. Note the higher peak values for Hit trials than CR1 trials. See also Table S1.

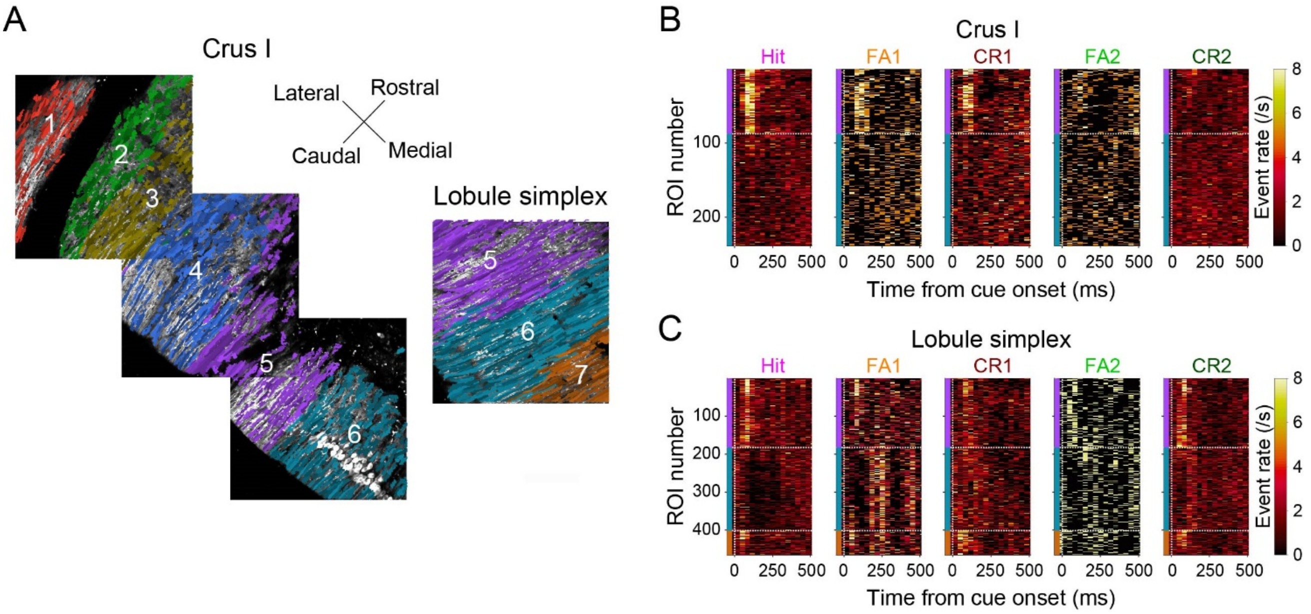

Figure S5. Zonal assignment across lobules and lobular specializations in task-related complex spike signals. Related to **Figure 4**

(A) Assignments of functionally-defined zones in Crus I and lobule simplex across fields of view (mouse 1 in Figure 4). Zones in lobule simplex are colored on the basis of spatial alignment of zones in Crus I. Note that a new zone 7 (orange) was identified in lobule simplex. (B) Trial averaged event rate heat map of ROIs from the most medial field of view in Crus I (zones 5 and 6) for each trial type. Vertical white dotted lines represent the onsets of sensory stimuli, and horizontal white dotted lines represent a boundary between zones. Zonal identity is represented by the thick colored bars on the left. (C) Same as (B) but for lobule simplex (zones 5–7). Note the similarity in alternating organization (zones 5 and 7, but not 6 are highly responsive to the task) but difference in sensory responsiveness (less response to air puff (FA1 and CR1 trials) and quicker and more responses to tone (FA2 and CR2 trials)).

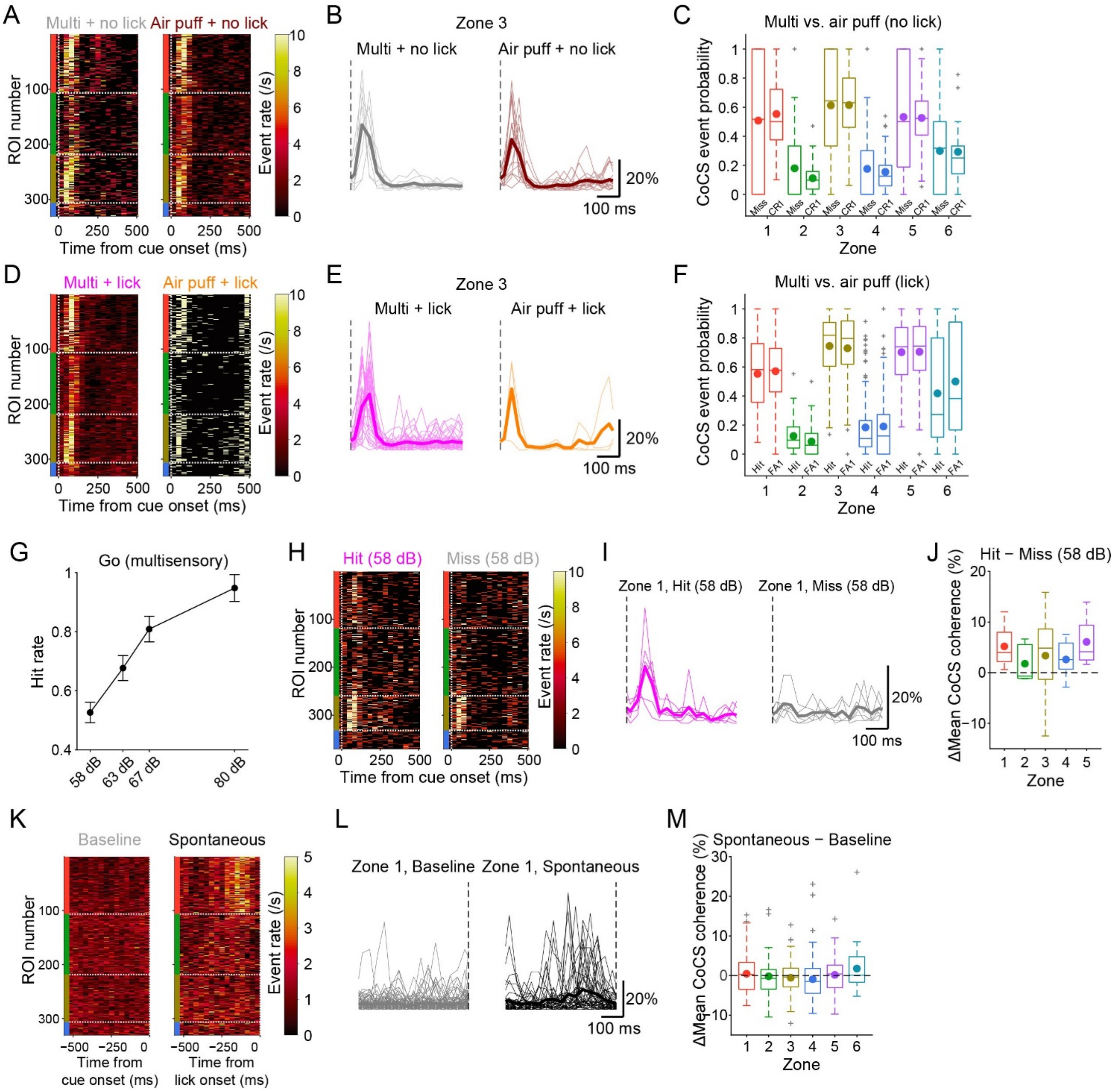

Figure S6. Zonal complex spike signals for multisensory stimuli are similar to those for somatosensory stimuli. Related to Figure 5

(A) Trial averaged event rate heat map of ROIs for Miss and CR1 trials from an example session. Vertical white dotted lines represent the onsets of the sensory cues and horizontal white dotted lines represent boundaries between zones. Zonal identity is represented by the thick colored lines on the left (zones 1–4). (B) Single trial co-activation traces of zone 3 for Miss (Multi + no lick) and CR1 (Air puff + no lick) trials in (A). Thin colored lines represent single trials and thick lines represent trial average (n = 8 and 17 trials for Miss and CR1). Vertical dotted lines represent the sensory cue onset. (C) Probability of co-activation (CoCS) events in zones 1-6 during Miss and CR1 trials in single sessions pooled across mice (N = 4 mice; 38, 38, 60, 78, 59, and 19 sessions for zones 1–6). Lines within boxes represent median, edges are interquartile range, and dots represent mean. Gray crosses represent outliers. Two-way ANOVA with repeated measures followed by post-hoc Tukey's test. (D) Same as (A), but for Hit (Multi + lick) and FA1 (Air puff + lick) trials. (E) Same as (B), but for Hit and FA1 trials (n = 38 and 3 trials for Hit and FA1). (F) Same as (C), but for Hit and FA1 trials (N = 4 mice; 82, 82, 148, 184, 144, and 42 sessions for zones 1–6). (G) Hit rate after multisensory stimuli (Go cue) with various levels of tone

(58, 63, 67, and 80 dB) during tone-attenuated sessions (n = 4 sessions from 4 mice). Dots and error bars represent mean ± S.E.M. (H) Trial-averaged event rate heat map of ROIs during Hit and Miss trials after multisensory stimuli with 58 dB tone from an example session. Horizontal white dotted lines represent boundaries between zones. Zonal identity is represented by the thick colored bars on the left (zones 1–4). (I) Single trial co-activation traces from zone 1 during Hit and Miss trials after multisensory stimuli with 58 dB tone. Thin colored lines represent single trials, and a thick line represents trial average (n = 6 and 6 trials for Hit and Miss). Vertical dotted lines represent the onset of sensory stimuli. (J) Differences in coherence level within co-activation (CoCS) events in zones 1–5 between Hit and Miss trials after multisensory stimuli with 58 dB tone in single sessions pooled across mice (N = 3 mice; 5, 5, 7, 9, and 5 sessions for zones 1–5). (K) Trial or epoch-averaged event rate heat map of ROIs before the cue onset (Baseline) and spontaneous lick bout onset (Spontaneous) from an example session. Horizontal white dotted lines represent boundaries between zones. Zonal identity is represented by the thick colored lines on the left (zones 1–4). (L) Single trial co-activation traces of zone 1 before sensory stimuli (Baseline, gray) and first licks of spontaneous lick bouts (Spontaneous, black). Thin colored lines represent single trials or epochs, and thick lines represent trial or epoch-average (n = 86 trials and 44 epochs for Baseline and Spontaneous). Vertical dotted lines represent the onset of sensory stimuli or first lick. (M) Differences in coherence level within CoCS events in zones 1–6 between spontaneous licking and baseline in single sessions pooled across mice (N = 4 mice; 46, 41, 92, 92, 93, and 28 sessions for zones 1–6). Note no enhancement in coherence before spontaneous licking. (C, F, J, and M) Lines within boxes represent median, edges are interquartile range, and dots represent mean. Gray crosses represent outliers. (C and F) Two-way ANOVA with repeated measures followed by post-hoc Tukey's test. (J and M) One sample t-test followed by Benjamini Hochberg correction. See also Table S1.

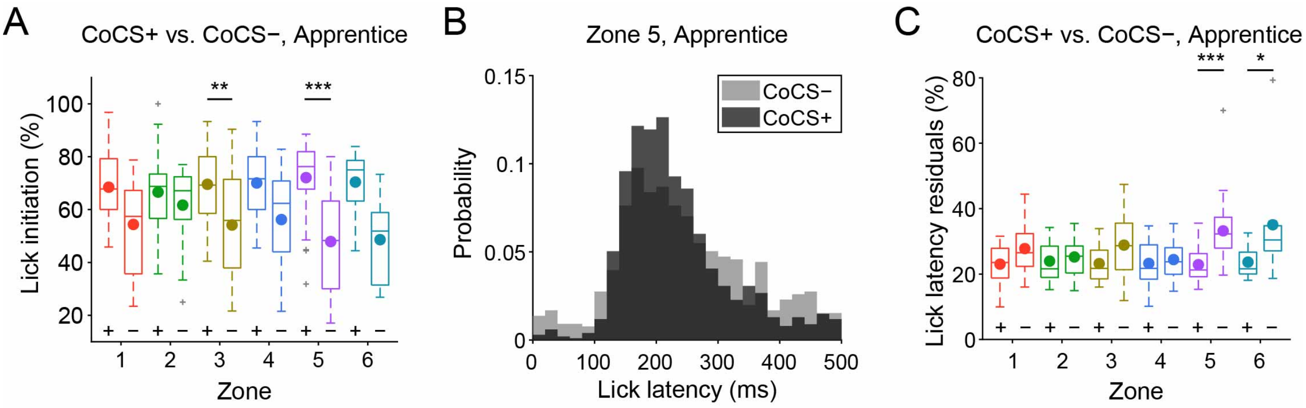

Figure S7. Coherent complex spike signals result in temporally precise motor initiation even during early stages of learning. Related to Figures 6 and 7

(A) Probability of lick initiation with or without co-activation (CoCS) events in zones 1–6 for all trials in single sessions at early learning stage pooled across mice (N = 4 mice; 22, 15, 34, 26, 29, and 9 sessions for zones 1–6). Lines within boxes represent median, edges are interquartile range, and dots represent mean. Gray crosses represent outliers. (B) Distribution of the latency of first lick in the licking trials (Hit, FA1, and FA2 trials) with or without co-activation (CoCS) events in zone 5, pooled across mice (N = 4 mice; 1,013 and 656 trials). (C) Same as (A), but for lick latency residuals in licking (Hit, FA1, and FA2) trials (N = 4 mice; 21, 13, 35, 24, 28, and 7 sessions for zones 1–6). (A and C) *$p < 0.05$; **$p < 0.01$; ***$p < 0.001$; two-way ANOVA with repeated measures followed by post-hoc Tukey's test. See also Table S1.
